# Unraveling the disease pyramid: the role of environmental micro-eukaryotes in amphibian resistance to the deadly fungal pathogen *Batrachochytrium dendrobatidis*

**DOI:** 10.1128/msystems.01436-25

**Published:** 2025-12-15

**Authors:** Rayan Bouchali, Hugo Sentenac, Kieran A. Bates, Matthew C. Fisher, Dirk S. Schmeller, Adeline Loyau

**Affiliations:** 1 Toulouse INP, CNRS, IRD, CRBE, Université de Toulouse 27091, Toulouse, France; 2 Chrono-environnement (UMR 6249), CNRS, Université Marie et Louis Pasteur 27000, Besançon, France; 3 Faculty of Medicine and Dentistry, Blizard Institute, Queen Mary University of London 105711, London, United Kingdom; 4 Department of Infectious Disease Epidemiology, School of Public Health, Imperial College London 156430, London, United Kingdom; Syracuse University, Syracuse, New York, USA

**Keywords:** high mountain lakes, disease pyramid, microbial ecology, disease ecology, amphibian immunity, 18S rRNA gene, chytrid fungus, Bayesian models

## Abstract

The disease pyramid conceptualizes the predictors of host infection risk, linking the host, the pathogen, environmental conditions, and both host and environmental microbiomes. However, the importance of the interaction between environmental and host-associated microbiomes in shaping infectious disease dynamics remains poorly understood. While the majority of studies have focused on bacteria, the role of micro-eukaryotes has been seldom investigated. Here, we explore three axes of the disease pyramid using an 18S rRNA gene metabarcoding approach to analyze the micro-eukaryotic assemblages of biofilm, water, and skin samples from three European amphibian species. Skin bacterial communities of the investigated amphibian populations have already been shown to be impacted by the presence of the lethal fungal pathogen *Batrachochytrium dendrobatidis* (*Bd*), with a higher abundance of protective bacteria in infected populations and a greater environmental microbial contribution to the skin microbiota in *Bd*-positive lakes. Here, we explored the relationships between the micro-eukaryotic skin communities of these tadpole populations with their surrounding environment. Tadpoles were sampled at 22 mountain lakes located in the Pyrenees (France), 8 of which harbored amphibian populations infected by *Bd*. We found that biofilms from *Bd*-negative lakes had higher environmental micro-eukaryotic diversity and a greater abundance of putative anti-*Bd* fungi, both in the environment and on the skin microbiota of *Bufo spinosus* and *Rana temporaria*, but not of *Alytes obstetricans*. Bayesian SourceTracker analysis further showed that the environmental contribution from biofilms to amphibian skin micro-eukaryotic assemblages was higher in *Bd*-positive lakes for *B. spinosus* and *R. temporaria*, but not for *A. obstetricans*.

**IMPORTANCE:**

Research on host-associated microbiomes and infectious diseases has mostly focused on bacteria, overlooking the potential contributions of micro-eukaryotes to infection dynamics. Here, we show that environmental and skin-associated micro-eukaryotes—especially putative anti-*Batrachochytrium dendrobatidis* (*Bd)* fungi—differ between *Bd*-positive and *Bd*-negative amphibian populations in mountain lakes. Our results suggest that micro-eukaryotes influence disease resistance and microbiome assembly, similarly to bacteria. Importantly, environmental reservoirs of micro-eukaryotes appear to contribute differently across infection contexts. These findings demonstrate the importance of adopting a broader microbiome perspective that includes micro-eukaryotes when investigating the ecological mechanisms underlying infectious disease risk.

## INTRODUCTION

Since the role of the host-associated microbiome in disease dynamics has been recognized (1–3), its contribution to host immunity has become increasingly evident (4–6). In humans, exposure to less diverse environmental microbiomes is associated with reduced immune effectiveness, underscoring the vital role of environmental microbes (7). Yet, the role of environmental microbiomes remains little explored in natural settings, despite their potential importance both as a biological barrier limiting pathogen spread in the environment and as a reservoir of beneficial microbes that, once colonizing the host, enhance immunity and resilience (8, 9). This knowledge gap has led to the concept of the disease pyramid, an extension of the classical disease triangle (including host, pathogen, and environment). In the disease pyramid, host- and environment-associated microbiomes are considered as an additional dimension influencing disease outcome. The term “pyramid” highlights this expansion from three (triangle disease) to four interacting components, rather than implying a hierarchical organization, and underscores their combined role at both individual and population levels (10–13). It has indeed been well established that populations are much less prone to outbreaks of infectious diseases when the ecosystem harbors micro-organisms that can inhibit the pathogen directly in the environment (14) and/or hamper the colonization by the pathogen through direct competition (15) or predation (16). The environmental microbiome can also promote host immunity by transferring communities of protective microbes to the host through coalescence processes (9).

Amphibians are one of the most well-studied vertebrate models for unraveling interactions between hosts, pathogens, the environment, and both host and environmental microbiomes. Interest in amphibians and their skin microbiome has emerged in the face of the global decline of this vertebrate group due to the panzootic chytridiomycosis, a skin disease caused by the fungal pathogen *Batrachochytrium dendrobatidis* (*Bd*) (17, 18). The amphibian skin microbiome is well known to contribute to host immunity by harboring protective bacterial communities (1, 19). For example, bacterial community structure and the relative abundance of protective bacteria have been shown to differ based on *Bd* exposure in amphibian populations (8), and some of the protective bacteria were found to come from the surrounding environment, such as biofilm, water, or sediments (5, 20, 21). Water was shown to contribute more to the amphibian skin bacterial communities in comparison to biofilm, and this contribution was higher in populations infected by *Bd* (R. Bouchali, H. Sentenac, D. S. Schmeller, A. Bernardo-Cravo, and A. Loyau, unpublished data).

Most prior studies seeking to unravel the interactions depicted in the disease pyramid have focused on bacteria only. In contrast, micro-eukaryotes, comprising fungi, protists (including apicomplexans), and other small microscopic eukaryotic organisms, have been largely overlooked and remain the least studied microbial paraphyletic group, with only 5% of metabarcoding projects devoted to their diversity and ecology (22). While representing only 1%–5% of total microbial cells, the functional roles of micro-eukaryotes should not be underestimated, as they are important metabolite producers (23) and typically have much larger cell sizes than prokaryotes, enabling single organisms to occupy relatively large surface areas. Micro-eukaryotes also play key roles in cross-kingdom interactions. For example, certain taxa promote the diversity of favorable bacteria and, in the human gut, enhance microbiome diversity by preventing the colonization of dominant taxa (24). At the microscale, food webs involve predation among micro-eukaryotes as well as on bacteria, processes that can shape microbial community dynamics and composition across all microbial kingdoms (25). More specifically, yeasts may also exhibit anti-toxin properties against pathogenic bacteria such as *Vibrio cholerae* and harbor anti-bacterial and anti-viral functions that are important in shaping the microbial communities of the human gut (23). Micro-eukaryotes may also interact directly with host immunity; for example, the protozoan *Tritrichomonas musculis* can decrease the risk of bacterial infection in a vertebrate host by activating the inflammasome (26).

Studies on the micro-eukaryotic communities inhabiting amphibian skin are very scarce, mainly focusing on fungal communities (27). In amphibians, fungi are indeed the most abundant micro-eukaryotes, making up 60% of total eukaryotic cells (28). Fungi have also been found to be highly efficient in inhibiting *Bd*, with almost half of the amphibian skin mycobiome showing anti-*Bd* properties *in vitro* (29), including the isolates belonging to the *Neobulgaria* and *Pleosporales* genera (30). However, fungal communities colonizing amphibian skin did not differ according to *Bd* infection status, but infection was correlated to specific fungal operational taxonomic units (31). While environmental micro-eukaryotic communities can directly influence pathogen survival, host microbiota, and thus host health, their broader function within the disease pyramid is not understood. Some micro-eukaryotic organisms have shown anti-*Bd* properties, especially ciliates, with one study demonstrating a reduction in *Bd* zoospore abundance driven by predatory ciliates, such as *Paramecium* and *Spirostomum* (16). Ciliates residing, even temporarily, on the skin may also be key players in colonization resistance against *Bd*. Many larval amphibians also extensively feed on environmental biofilms (32), which are crucial for aquatic ecosystems and harbor many micro-eukaryotes (33). The latter might positively influence host health through multiple ways, which remain largely unknown: by containing vital nutrients (34), by inactivating/consuming *Bd* motile zoospores (14), and by contributing to the enrichment of the skin and gut host microbiome through coalescence processes.

Here, we used 18S rRNA metabarcoding and the latest microbiome analysis tools to investigate the roles of both environmental and host-associated micro-eukaryotes in driving resistance to Bd in high mountain lakes of the Pyrenees. First, we assessed the importance of environmental micro-eukaryotic assemblages in host protection against *Bd* in natural populations of three amphibian species. Second, we infer functional anti-*Bd* abilities in amphibian skin, biofilm, and water (planktonic) samples. Specifically, we tested the following hypotheses: (i) the environmental microbiome acts as a biological barrier against *Bd*, reflected by a greater prevalence and abundance of putative anti-*Bd* microorganisms in pathogen-free lakes, and (ii) there is a close relationship between the amphibian skin and the environmental microbiome through transfers and engraftment of putative anti-*Bd* micro-eukaryotic cells.

## RESULTS

### Micro-eukaryotic communities of amphibian tadpole skin and their living environment

#### Micro-eukaryotic diversity and richness

For all compartments, we did not detect an effect of sampling events (i.e., season and year) on the α-diversity (ANOVA, all *P* > 0.01). Biofilm samples showed the highest value for Shannon and Simpson indexes, indicating a higher diversity with both dominant and rare species, as well as for amplicon sequence variant (ASV) richness (ANOVA, all *P* < 0.01; Fig. S1; Tables S1 and S2). Skin micro-eukaryotic communities had a low diversity and richness (richness: from 11 to 348 ASVs; Shannon: 0.37–4.80; Simpson: 0.13–0.98; evenness: 0.1–1), with significant dissimilarities between amphibian species (Fig. S1; Tables S1 and S2).

The β-diversity was significantly different in the ASV profiles of biofilm and water samples (PERMANOVA, *R*^2^ = 0.59, *P* < 0.01; Fig. S2), as well as in regard to the lake of origin (PERMANOVA, *R*^2^ = 0.86, *P* < 0.01). The β-diversity of amphibian skin was not different from either water or biofilm microbiomes (biofilm: PERMANOVA: *R*^2^ < 0.01, *P* = 0.43; water: PERMANOVA: *R*^2^ = −0.02, *P* = 0.98). It was determined by the lake of origin (PERMANOVA, *R*^2^ = 0.21, *P* < 0.01) and not species-specific (*Envfit* model adjusted with the lake effect: *R*^2^ = 0.01, *P* > 0.05).

We compared the richness and diversity of micro-eukaryotic (18S ASVs) and bacterial (16S ASVs) skin communities from the same amphibian population, using 16S data from a previous study (8). Only ASV richness (linear regression, all *P* < 0.05) was correlated between bacterial and micro-eukaryotic communities (Fig. S3). For biofilm and water, we found a significant congruence between the ordinations of the bacterial and micro-eukaryotic communities (protest, *m*^2^ = 0.16, *r* = 0.92, *P* = 0.001) (Fig. S4a). For amphibian skin communities, significant congruence between 16S and 18S ordinations was detected in all three amphibian species (*Alytes obstetricans: m*^2^ = 0.83, *r* = 0.41, *P* = 0.011; *Bufo spinosus: m*^2^ = 0.15, *r* = 0.92, *P* = 0.001; *R. temporaria: m*^2^ = 0.66, *r* = 0.59, *P* = 0.001, Fig. S4b).

#### Micro-eukaryotic and protective taxa inhabiting the amphibian tadpole skin and their environment

**Fig 1:**
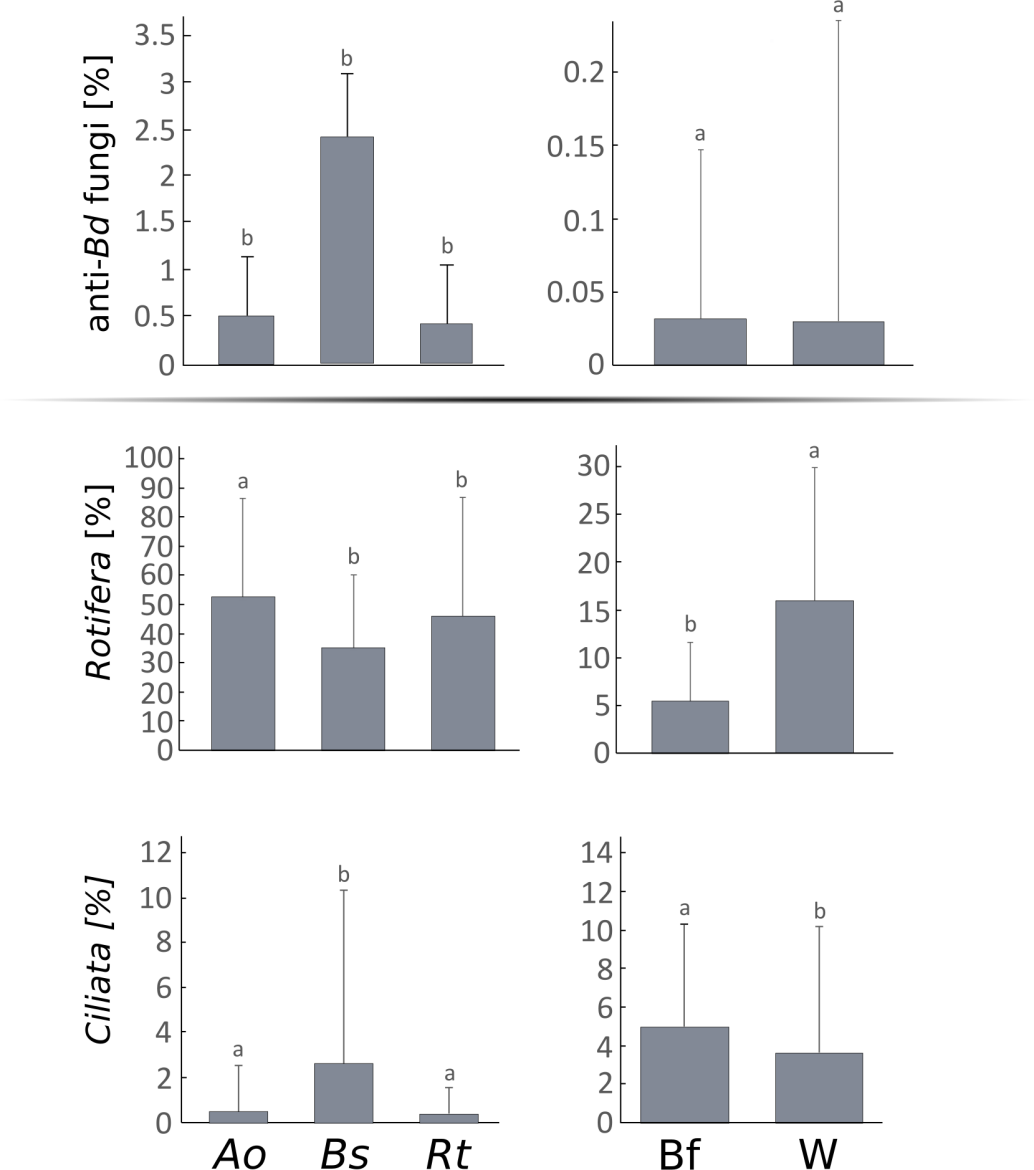
Relative abundance of anti-*Bd* fungi, *Ciliata,* and *Rotifera* in samples from the skin of three amphibian species, environmental biofilm, and water. The different letters indicate statistical groups that are significantly different from each other (ANOVA test followed by Tukey’s *post hoc* test, *P* < 0.05).

We characterized the most abundant micro-eukaryotic taxa in amphibian skin, water, and biofilm samples (Table S3). In all amphibian species, *Vorticella* dominated skin microbiomes, but we also observed species-specific enrichment of genera (ANCOM-BC, all *P* < 0.05, Fig. S5). Dominance of ASVs was also different between the amphibian species, biofilm, and water (Tables S4 and S5; Fig. S5). The five most abundant genera in biofilms were generally rare in water (<0.5%), and conversely, the five most abundant genera in water were rare in biofilms. Characterization of micro-eukaryotic taxa in amphibian skin, biofilm, and water samples allowed detection of four putative anti-*Bd* fungal genera: *Aspergillus*, *Basidiobolus*, *Cyberlindnera*, and *Penicillium*. Only *Aspergillus* and *Penicillium* were detected on skin (*A. obstetricans*: 0.07% and 0.41%; *B. spinosus*: 0.08% and 2.33%; and *R. temporaria*: 0.09% and 0.29%), with no significant differences between species (ANOVA, *P* > 0.05; Fig. 1; Table S6). In biofilm and water, the four genera were rare (*Penicillium* 0.01% and 0.02%; *Basidiobolus* 0.01% and <0.01%; *Aspergillus* <0.01% and 0.00%; *Cyberlindnera* < 0.01%; ANOVA, *P* > 0.05; Table S6). *Ciliata* and *Rotifera* were abundant on tadpole skin and in the environment, with higher *Rotifera* proportions in *A. obstetricans* and water, and higher *Ciliata* proportions in *B. spinosus* and biofilms (ANOVA, *P* < 0.01; Table S6). Five *Ciliata* genera harboring anti-*Bd* species (*Blepharisma*, *Dileptus*, *Euplotes*, *Paramecium*, and *Spirostomum*) showed comparable abundances across hosts, water, and biofilms (ANOVA, *P* > 0.05; Table S6).

### Micro-eukaryotic assemblage and *Bd* infections

#### Relationships between *Bd* and the micro-eukaryotic diversity and richness

**Fig 2:**
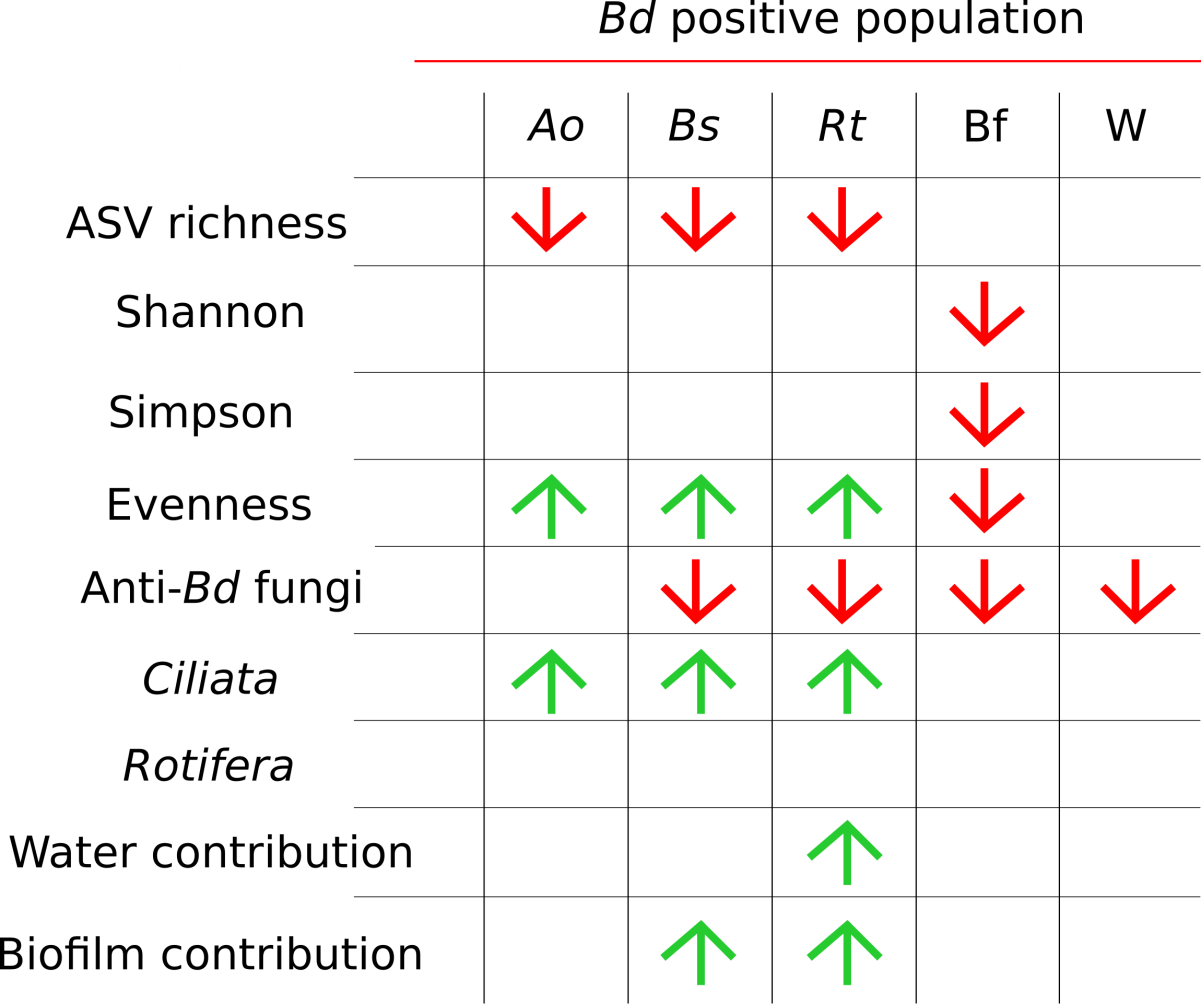
Impact of the presence of *Bd* on the micro-eukaryotic communities of amphibian skin, biofilm, and water compartments, showing the significant increase (green) or decrease (red) of the micro-eukaryotic parameter values. *Ao, A. obstetricans; Bs, B. spinosus; Rt, R. temporaria*; Bf, biofilm; and W, water.

The α-diversity of tadpole skin and biofilm, but not water, differed significantly between *Bd*-positive and *Bd*-negative lakes (Fig. 2; Fig. S6 and S7; Tables S1, S2, and S7). ASV richness was lower in *Bd*-positive lakes for *A. obstetricans*, *B. spinosus*, and *R. temporaria* (Wilcoxon test, all *P* < 0.01) (Fig. S6; Tables S2 and S7), whereas Shannon and Simpson diversity on skin did not differ (Wilcoxon test, all *P* > 0.05) (Table S7). In biofilms, micro-eukaryotic diversity and evenness were higher in *Bd*-negative lakes (Shannon: *W*_46_ = 4,116, *P* < 0.01; Simpson: *W*_15_ = 3,969, *P* < 0.01; evenness: *W*_34_ = 3,727, *P* < 0.05), while ASV richness showed no significant difference (*W*_50_ = 3,635, *P* > 0.05). No differences with regard to the infection status of the amphibian population were observed in water micro-eukaryotic communities (all *P* > 0.05; Fig. S6; Table S7).

The β-diversity of micro-eukaryotic assemblages was not different between amphibian skin samples from *Bd-*positive and *Bd*-negative lakes (PERMANOVA, *R*^2^ = 0.01, *P* = 0.33), but significant differences were apparent in biofilm (PERMANOVA, *R*^2^ = 0.15, *P* < 0.01) and water community compositions (PERMANOVA, *R*^2^ = 0.08, *P* < 0.01) (Fig. S2).

#### Relation between *Bd* presence and the abundance of protective micro-eukaryotic taxa

**Fig 3:**
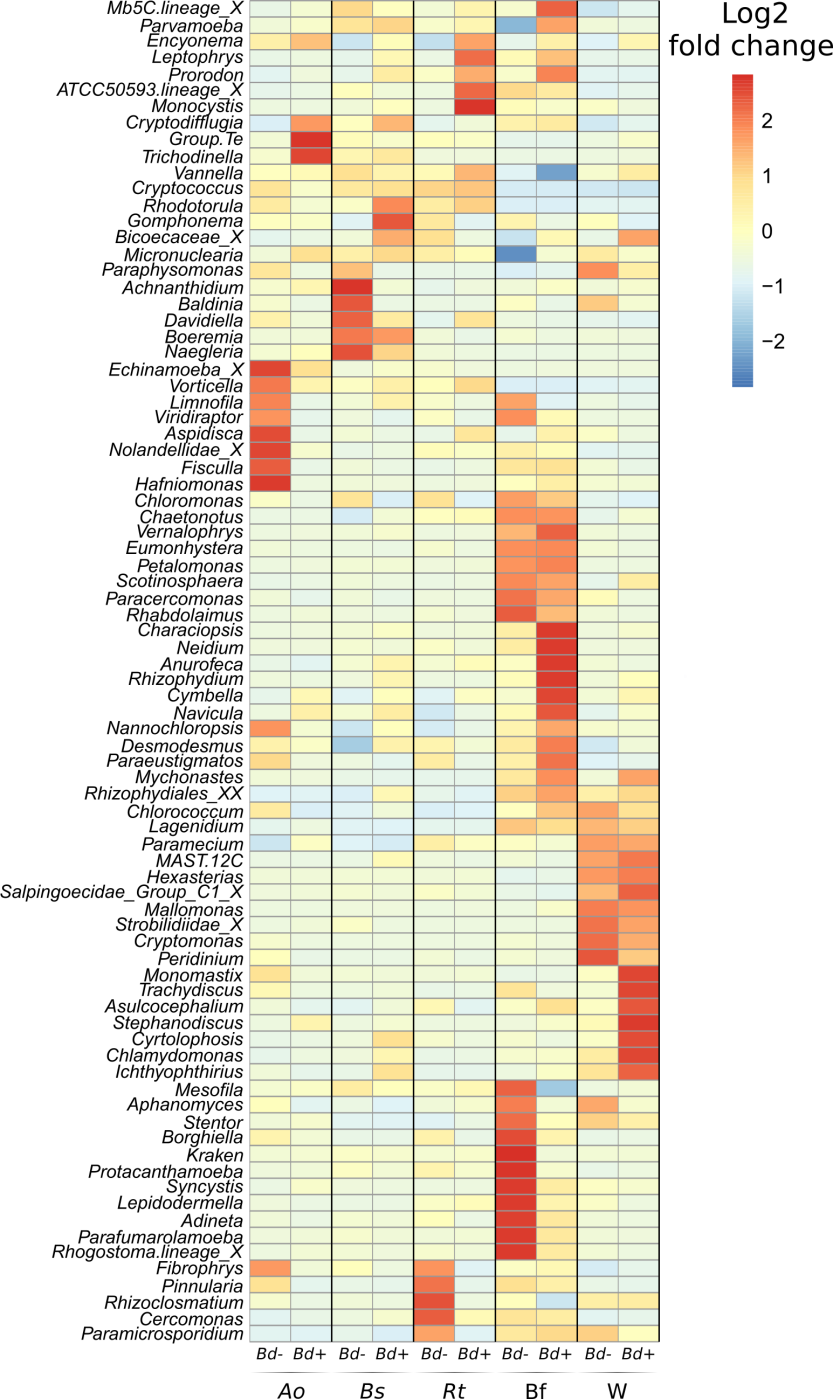
Heatmap of the micro-eukaryotic genera differently associated with the host species, the biofilm, and the water samples, and according to the *Bd* infection status (negative vs positive), as revealed by the analysis of compositions of microbiomes with bias correction (ANCOM-BC). When the genus was unknown, the family (_X) or the order (_XX) is indicated. Only taxa with a significant overall distribution bias are shown.

**Fig 4:**
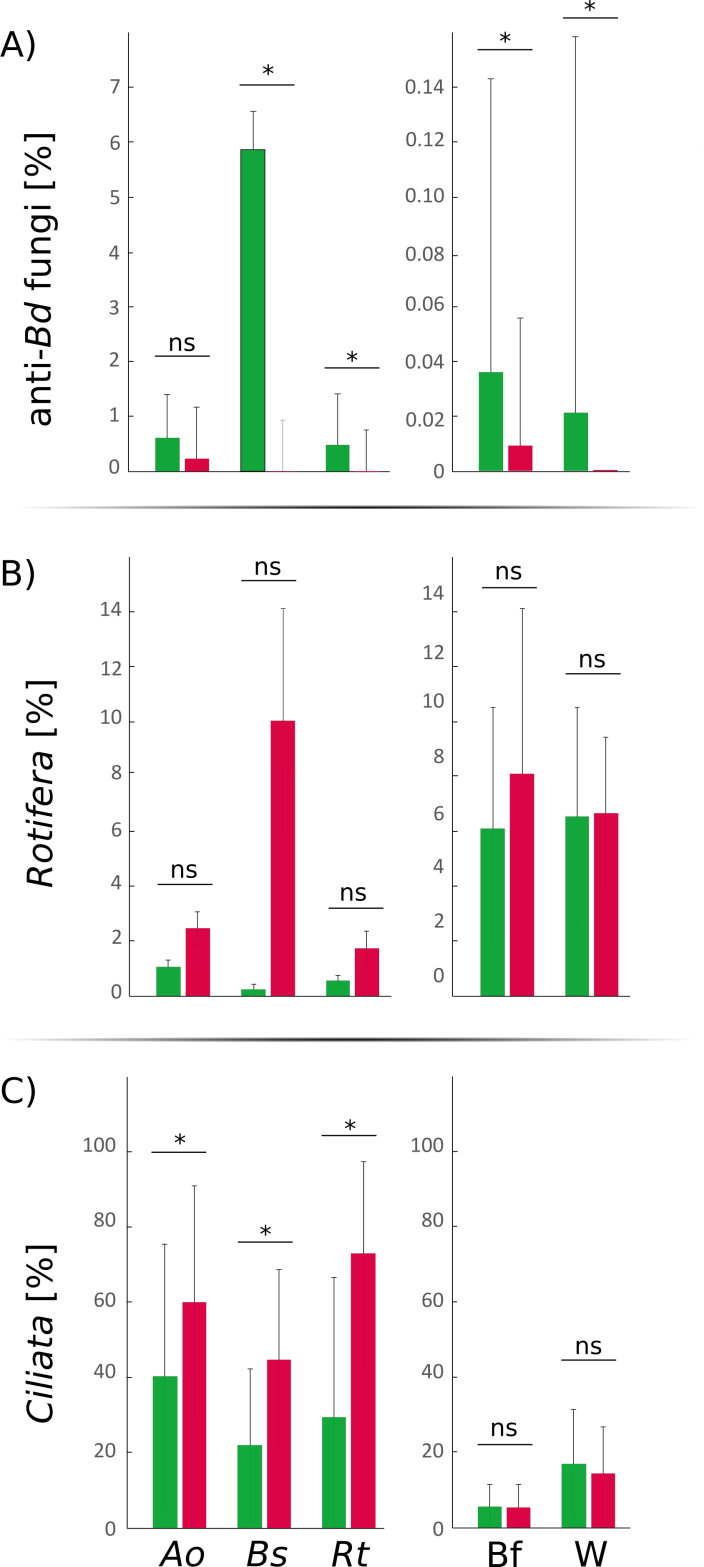
Average relative abundance of the (**A**) putative anti-*Bd* fungi, (**B**) *Rotifera,* and (**C**) *Ciliata* according to the host species and the *Bd* infection status of the considered lakes (green, *Bd* negative; red, *Bd* positive). Asterisks highlight significant differentiation between *Bd*-positive and *Bd*-negative lakes (Wilcoxon test). *Ao, A. obstetricans; Bs B. spinosus, Rt, R. temporaria*; Bf, biofilm; and W, water.

Genera differentially distributed according to *Bd* infection status were identified for all compartments using ANCOM-BC (Fig. 3). None of the genera showing differentiated repartitions were putative anti-*Bd* taxa; however, the total abundance of putative anti-*Bd* micro-eukaryotes was associated with *Bd* presence (Fig. 2 and 4). In *B. spinosus* and *R. temporaria*, but not *A. obstetricans*, putative anti-*Bd* fungi were more abundant on skin from *Bd*-negative lakes (5.82%, 0.47%, and 0.59%, respectively) than *Bd*-positive lakes (0.00%, 0.01%, and 0.36%, respectively) (Wilcoxon test, *W*_9_ = 45, *P* < 0.05; *W*_113_ = 2, *P* < 0.05; and *W*_113_ = 0.45, *P* > 0.05; Fig. 2 and 4A; Table S8). Ciliata were more abundant on amphibian skin from *Bd*-positive lakes across all species (Wilcoxon test, all *P* < 0.05; Fig. 4C; Table S8), while *Rotifera* showed no differences (all *P* > 0.05; Fig. 4B). Biofilm and water samples had higher putative anti-*Bd* fungi in *Bd*-negative lakes, but *Bd* presence was not linked to *Rotifera* or *Ciliata* abundance (Wilcoxon test, all *P* > 0.05; Fig. 4; Table S8). Relative abundance of putative anti-*Bd Ciliata* genera did not differ with *Bd* infection, except for *A. obstetricans*, which was higher in *Bd*-positive lakes (*W*_7_ = 3,570, *P* > 0.05; Table S8).

#### Coalescence processes linked to *Bd* infection status

**Fig 5:**
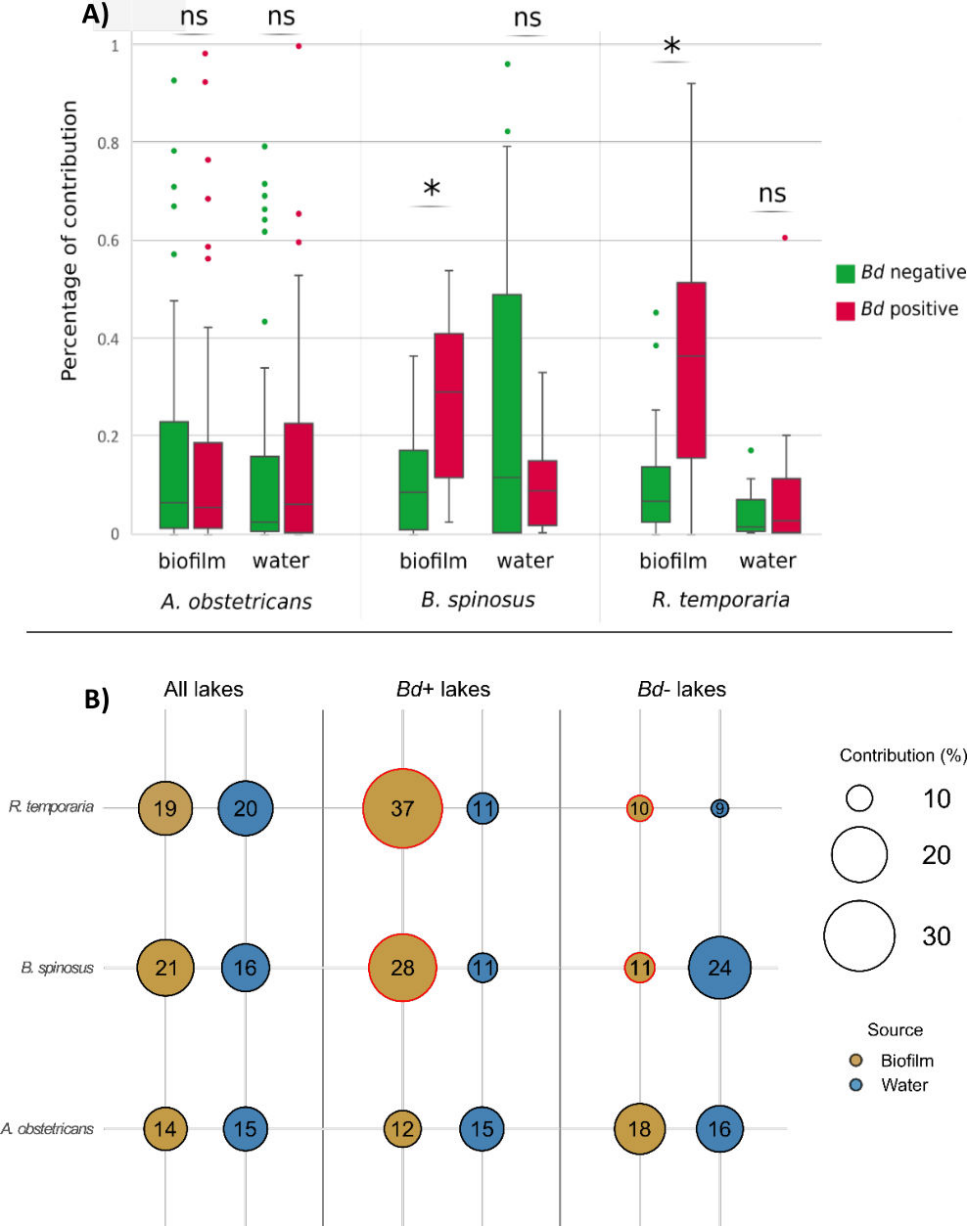
(**A**) Boxplots showing the inferred percentage of contribution from biofilm and water samples to the assembly of amphibian skin micro-eukaryotic communities, as estimated by Bayesian SourceTracker analysis. (**B**) Mean inferred environmental contributions to amphibian skin communities. Asterisks and red circles denote significant differences in contributions between *Bd*-positive and *Bd*-negative lakes (Wilcoxon test).

The SourceTracker analysis revealed that environmental biofilm and water communities contributed significantly to the skin micro-eukaryotic communities of *A. obstetricans* (14 % ± 19% and 14%  ±  21%), *B. spinosus* (20% ± 16% and 16% ± 20%), and *R. temporaria* (19% ± 21% and 19% ± 30%) (Tables S9 and S10). *Bd* infection status influenced these environmental transfers (Fig. 2 and 5; Tables S9 and S11). Biofilm contributions were higher in *Bd*-positive lakes than *Bd*-negative lakes for *R. temporaria* (37% ± 25% vs 9% ± 10%; *W*_20_ = 130, *P* < 0.01) and *B. spinosus* (27% ± 15% vs 10% ± 10%; *W*_9_ = 26, *P* < 0.01), but not for *A. obstetricans* (12% ± 16% vs 18% ± 23%; *W*_67_ = 4,112, *P* > 0.05). Water contributions to skin microbiota did not differ with Bd infection status (Wilcoxon, all *P* > 0.05; Fig. 5; Table S11).

A phylogenetic null model showed that micro-eukaryotic communities of amphibian skin are driven mostly by stochastic processes, with low average absolute βNTI values (<1) and high variability between individuals. The presence of *Bd* did not affect the assembly processes, with similar βNTI values between amphibian skin from *Bd*-positive and *Bd*-negative lakes (ANOVA, *F*_7_ = 1.422, *P* = 0.20).

## DISCUSSION

Our study examined the links between micro-eukaryotic communities on tadpole skin and environmental sources, as well as their relationship to the presence of the fungal amphibian pathogen *Bd*. Our study builds on recent experimental evidence showing that environmental biofilms can inactivate *Bd* zoospores (14), suggesting that the environmental microbiome could prevent the invasion of ecosystems by the pathogen. Our metabarcoding approach provides ecological context to these mechanistic findings, integrating amphibian skin, biofilm, and water microbiomes. While metabarcoding alone does not establish causality, it allows us to situate field-based observations within the framework of the disease pyramid (11) by identifying associations between microbial assemblages and *Bd* occurrence. Our results show that environmental and amphibian skin micro-eukaryotic communities differed with the *Bd* infection status of the studied amphibian populations. Biofilm α-diversity, but not water α-diversity, was reduced in infected lakes, suggesting that the lower diversity of micro-eukaryotic communities in biofilms from infected lakes may have favored the environmental invasion by *Bd*.

The biotic resistance theory suggests that a rich biodiversity and its associated interactions (e.g., competition and predation) could prevent the invasion of undesirable species (35). Here, rich and diverse biofilms may be more stable and more resistant to perturbation, increasing the probability of inactivation of *Bd* zoospores, the infectious stage of *Bd* (i.e., biological barrier) (14, 36). The dominance of some species (i.e., low evenness) can lead to a decline in microbial interactions and metabolic and mechanistic functions that are crucial for inhibiting pathogen establishment in the environment (37). In biofilms from *Bd*-positive lakes, the lower community diversity may reduce the inhibitory priority effect (38, 39), leaving ecological niches vacant and open to pathogen invasion (40). Conversely, in richer biofilms, as observed in *Bd*-negative lakes, established micro-eukaryotes may influence community assembly by occupying niches and using resources, thereby reducing the opportunities of establishment for newly arriving species, including emerging pathogens (41). A richer biofilm also increases the probability of containing protective micro-eukaryotes, including *Bd*-inhibitory ones, by increasing the diversity of functions (42). Our results also suggest that, in addition to excluding *Bd* indirectly by eliminating available niches, micro-eukaryotic communities may also be able to directly inhibit the pathogen in the environment and prevent its installation, for example, by the production of anti-fungal compounds or by predation.

On the tadpole skin, we did not observe higher abundances of anti-*Bd* fungi in *Bd*-positive lakes, but *Ciliata* were generally more abundant. *Ciliata* are unicellular microscopic predators that can feed on lipid-rich *Bd* zoospores (16), but they are generally less versatile compared to fungi (i.e., they are more specialized and less able to use a variety of nutrient sources). They can rapidly proliferate in response to a sudden increase in a specific resource (43) and may benefit from the presence of *Bd* on the skin of amphibian tadpoles, which represents a significant nutrient source in oligotrophic environments (44). Infection by *Bd* and the induced dysbiosis in the skin microbiome (5, 45) could create a new ecological niche that *Ciliata* can exploit particularly well, similar to opportunistic fast-growing bacteria (8). *Ciliata* are more mobile compared to fungi and, therefore, are able to reach and colonize such a new niche, in our case, likely from biofilms on which tadpoles graze. Conversely, fungi are more versatile organisms and more efficient in oligotrophic settings as found in most mountain lakes (46), where they can exploit a broader range of nutrient sources and persist in the absence of *Bd*. Anti-*Bd* fungi may therefore be maintained through other interactions, including predation on other environmental microbes or utilization of alternative carbon sources, thereby contributing to a more stable and protective fungal community. Such a community may potentially prevent the establishment of *Bd* by consuming or otherwise inactivating its zoospores during colonization attempts. In contrast, in *Bd*-positive lakes, the proliferation of *Bd* and the associated dysbiosis could create conditions less favorable for fungi but more suitable for opportunistic protists such as ciliates.

The differentiated distributions of anti-*Bd* fungi and *Ciliata* illustrate the difficulty of investigating the ecological interactions between the host, the host microbiome, *Bd,* and environmental microbial communities, as depicted in the disease pyramid. The temporality of two processes, first the colonization of the emerging pathogen prevented (or not) by the environmental micro-eukaryotic communities, and second, the response of the amphibian skin microbiome once *Bd* is established in amphibian assemblages, is not easily disentangled. Amphibians can respond adaptively to *Bd* with their skin microbiome shifting over time, as suggested by the adaptive microbiome theory (5). A key question is whether this process favors the proliferation of *Ciliata* that are capable of predating upon *Bd*, thus enhancing protection against the pathogen. Such an increase in protective microorganisms can be followed by a decrease in richness and an increase in dominance of beneficial microorganisms (5). We indeed found a lower ASV richness on tadpole skin from *Bd*-positive lakes, which may reflect enrichment in protective micro-eukaryotes that hamper *Bd* infections. The impact of *Bd* on the enrichment of microbes from environmental sources was already observed for bacteria (R. Bouchali, H. Sentenac, D. S. Schmeller, A. Bernardo-Cravo, and A. Loyau, unpublished data). However, only planktonic aquatic bacteria were enriched within the amphibian skin tadpoles, whereas micro-eukaryotes from biofilms were enriched when *Bd* was present. Planktonic micro-eukaryotes are adapted to life in suspension (47). Their ability to adhere to surfaces is often weaker than that of organisms living in biofilms. Conversely, biofilm micro-eukaryotes are specialized in adhesion and growth on solid substrates. They often possess structures that facilitate their attachment to surfaces (e.g., filaments, cilia, and flagella). Links between *Bd* presence and bacterial or micro-eukaryotic transfers (from the environment to the amphibian skin) were only observed for *B. spinosus* and *R. temporaria* (R. Bouchali, H. Sentenac, D. S. Schmeller, A. Bernardo-Cravo, and A. Loyau, unpublished data), whereas the inferred contribution from environments to the skin microbiota of *A. obstetricans* was lower. Between these species, there is a marked difference in the life-history trait of brumation. *A. obstetricans* has overwintering tadpoles that can spend several winters in the lake before metamorphosis, while the other two species complete their cycle from egg to metamorph within the same season. The longer exposure of *A. obstetricans* to lake environmental microbial communities may create inhibitory effects that would not allow a rapid adaptation to changing pathogen exposure.

In a previous study, we showed an opposite response to *Bd* for bacteria colonizing amphibian skin, which originated more frequently from water (R. Bouchali, H. Sentenac, D. S. Schmeller, A. Bernardo-Cravo, and A. Loyau, unpublished data), while micro-eukaryotes may originate more frequently from biofilms. This suggests complementary mechanisms to shape the skin microbial communities that ultimately lead to a more or less effective protection against skin pathogens. Bacteria, micro-eukaryote communities, and other microbial organisms are interdependent (48), and some can recruit others, thus influencing their dynamics and ecological function (23), even within amphibian skin microbiota (49). Therefore, integrated approaches, based on holistic frameworks such as the disease pyramid, are needed to grasp the coupling between the structures and functions of microbial communities. These approaches would enable a deeper understanding of the diversity of interactions occurring in ecosystems and their roles in warranting resilience in the face of increasingly numerous perturbations. Such knowledge could then be leveraged to develop more general forms of ecosystem protection, built on nature-based solutions and reinforcement of ecosystem resilience.

## MATERIALS AND METHODS

### Study sites and sampling strategy

We sampled tadpole populations of three amphibian species, *Alytes obstetricans*, *Bufo spinosus,* and *Rana temporaria,* inhabiting 22 high mountain lakes (between 1,063 and 2,522 m above sea level) located in the French and Spanish Pyrenean Mountains (Europe) (Table S12). Our lake system covers an area spreading over 173 km from East to West and 16 km from South to North (2,768 km^2^). We focused on the tadpole skin microbiome because the larval stage is the point at which infection is established. Although tadpole skin is not fully keratinized and *Bd* primarily colonizes their keratinized mouthpart without lethality at this stage (50, 51), tadpoles can act as a reservoir that maintains and amplifies the pathogen in aquatic environments until metamorphosis, when susceptibility and mortality dramatically increase. Furthermore, the tadpole skin microbiome is of particular importance, as it seeds the metamorphic microbiome (52), which becomes critical when *Bd* shifts from colonizing mouthparts to keratinized skin during and after metamorphosis (50, 53, 54). Hence, the tadpole skin microbiome is of vital importance for resistance to infection later in development due to vertical transfer from tadpoles over metamorphs to adults (50, 53).

Five to ten tadpoles of each species were captured in the littoral zone close to the shore of each lake by dip netting. To investigate skin micro-eukaryotic communities, tadpoles were swabbed across their full body using a sterile dry swab (MW100, MWE Medical Wire, Corsham, UK). Mountain lakes were visited several times during early, mid, or late summer, two times in 2016, and three times in 2017 and 2018. In the same lakes, tadpoles were monitored for over 10 years for the presence of *Bd* (30 tadpoles were captured and sampled at each instance when present), and a qPCR was run in duplicate (8). Eight lakes had amphibian populations infected for at least 10 years (*Bd*-positive lakes), 12 had populations negative for *Bd* (*Bd*-negative lakes), and 2 had a changing infection status (defined as “changing”) (Table S12). Amphibian species showed different distributions: tadpoles of *A. obstetricans* were sampled across 13 lakes (in 2016, 2017, and 2018), *R. temporaria* across 15 lakes (only in 2017 and 2018), and *B. spinosus* across 3 lakes (only in 2018). This led to a total of 552 amphibian tadpoles swabbed, including 336 *A. obstetricans*, 164 *R. temporaria,* and 25 *B. spinosus*. Additionally, water samples were collected in the littoral zone where tadpoles live, using a clean Nalgene bottle disinfected with chlorhexidine and rinsed with physiological serum (NaCl 0.9%) and then lake water. Between 250 mL and 1 L of water, depending on the suspended matter in a given lake, were filtered with a 0.22 µm filter using a Nalgene filter holder and a vacuum hand pump. Biofilm was sampled by scraping rocks that were found at a depth of 15–30 cm, using a metal spatula that was disinfected with chlorhexidine and then rinsed with sterile physiological serum (NaCl 0.9%), and then put in a sterile 2 mL Eppendorf tube. All samples (swabs, biofilms, and filters) were immediately frozen on dry ice (−78°C) in the field and then stored at −25°C until extraction, which was usually performed the following month. This led to a total of 179 water and 203 biofilm samples (Table S12).

### 18S rRNA gene PCR amplification, sequencing, and metabarcoding pipeline

DNA from swabs was extracted with the Macherey-Nagel NucleoSpin Soil Kit (Valencia, CA, USA) according to the manufacturer’s protocol. Negative controls included the 0.22 µm filter, extraction kit, and PCR reagents. The V8–V9 regions of the 16S rRNA gene were amplified using V8f and 1510r primers (55, 56), with a PCR program of 3 min at 95°C, 30 cycles of 30 s at 55°C and 30 s at 72°C, and a final 5-min extension at 72°C. Amplicon quality was checked on a 1.5% agarose gel. Illumina MiSeq sequencing of PCR amplicons (2 × 250 bp) was performed by the GENOTOUL platform (Toulouse, France). Primers, linkers, and barcodes were removed with Cutadapt version 4.0 (Python 3.9.12) (57). Raw reads were processed with DADA2 version 1.26.0 (58) following the paired-end SOP (https://benjjneb.github.io/dada2/bigdata_paired.html). Briefly, reads were filtered with minLen = c(200, 200), truncLen = c(280, 260), maxN = 0, maxEE = c (5, 5), and truncQ = 2. Chimeras were removed using the consensus method from removeBimeraDenovo. ASVs were assigned taxonomy with SILVA version 138.1 using the Wang classifier (minimum bootstrap 80%) (59). Contaminant ASVs were removed with Decontam (prevalence method, threshold 0.5) (60). ASVs affiliated to large multicellular plants, such as *Embryophyceae*, and metazoans, such as *Vertebrata*, *Arthropoda*, *Platyhelminthes*, *Annelida,* and *Mollusca*, were discarded. The Decontam package did not detect contaminant ASVs. Samples with less than 1,000 reads and/or 10 ASVs were discarded. This led to a final data set of 16,717,680 reads and 26,536 ASVs across 294 *A. obstetricans*, 139 *R. temporaria*, 24 *B. spinosus*, 188 biofilms, and 156 water samples (see Table S12). The taxonomic affiliation of the resulting ASVs allowed the classification of 97% of the reads at the class level, comprising 1,006 genera (80% assignment rate) and 1,189 species (68%).

### Statistical analyses

#### Analysis of micro-eukaryotic richness and diversity

We first checked that not rarefying the micro-eukaryotic data set did not bias the results by comparing our unrarefied data with a data set rarefied to 1,010 reads. Rarefaction curves further indicated that sequencing depth was insufficient within the rarefied data set to capture the full micro-eukaryotic diversity, making rarefaction inappropriate (Fig. S7). Additionally, α-diversity indices were highly correlated (*R*^2^ = 0.99) between the rarefied and unrarefied data set, and beta-diversity analyses (Bray–Curtis, PERMANOVA) showed no significant differences (*R*^2^ < 0.0001, *P* = 0.168), supporting the use of unrarefied data.

All the statistical analyses were computed using the R software version 4.3.2. Data distributions were checked using the *check_distribution* from the R package Performance. Rarefaction curves were computed with the vegan R package version 2.6.4 (61) to check the sequencing depth (61) (Fig. S9). Micro-eukaryotic diversity (Shannon, Simpson, and Pielou’s evenness) and richness (ASVs number) indexes of amphibian skin and environmental samples, as well as β-diversity metrics (Bray–Curtis dissimilarity distances, PERMANOVA statistical tests, and NMDS), were computed with the vegan package. The chosen α-diversity indexes allowed a complementary insight into the microbial richness, with the Shannon diversity index being more sensitive to rare ASVs compared to the Simpson index, and Pielou’s evenness describing the homogeneity of the ASV distributions in each sample. We used Bray–Curtis dissimilarity as our main metric because it accounts for both the presence of taxa and their relative abundances, which are key to assessing host–microbe interactions and resistance to Bd. This choice is particularly relevant since *Bd* infection may alter the abundance of resident taxa rather than introducing new ones (8). PERMANOVAs were run using the following predictor variables: amphibian host species, environmental matrices (i.e., biofilm and water), and their combination with the *Bd* infectious status. ANOVAs and Tukey’s *post hoc* tests were used to compare the micro-eukaryotic richness and diversity indexes of amphibian skin samples according to the sampling season (early, mid, or late summer), as well as between amphibian species, and between biofilm and water. Due to a lower number of samples, we used Wilcoxon tests to compare the diversity indexes and richness between *Bd*-negative and *Bd*-positive lakes.

#### Comparison of the bacterial and micro-eukaryotic community richness and diversity

Bacterial sequences (16S rRNA) were extracted from accession number PRJEB46609 (ERP130803) and processed as described previously (8). α-diversity of 16S and 18S was compared using linear regression with the *lm* function of the R software. Linear regressions were illustrated with the ggplot2 version 3.5.1 package (62). β-diversity (Bray–Curtis ordinations) of 16S and 18S was compared using the *procrustes* function of the Vegan package performed on the NMDS ordinations. Significance of the congruence was tested using the Vegan *protest* function (permutation test, *n* = 999 permutations), a significant *P*-value meaning that there is a high similarity (=significant correlation) between the two microbial assemblages.

#### Characterization of the abundance of putative *Bd*-inhibitory micro-eukaryotes

Putative anti-*Bd* genera were identified using the database from Kearns et al. (29), which includes 45 genera shown to mitigate the infection of amphibians by *Bd*. We also analyzed the abundance of the *Ciliata* and *Rotifera* phyla, which are likely to reduce the abundance of *Bd* by predation (16, 63). This also allowed the characterization of putative anti-*Bd Ciliata* and *Rotifera* genera, which harbor species showing anti-*Bd* properties under experimental conditions. We used ANOVAs followed by a Tukey’s *post hoc* test to compare the abundance of these taxa between the different compartments and Wilcoxon tests to compare *Bd*-negative and *Bd*-positive lakes. In addition, we tested the differential abundance of genus between amphibian skin, biofilm, and water, according to the *Bd* infectious status, using the ANCOM-BC R package (64) (function *ancombc2*, FDR method of *P*-value adjustment).

#### Estimation of the micro-eukaryotic environmental contribution in the building of amphibian tadpole skin communities

The contribution of biofilm and water in the building of the micro-eukaryotic communities of amphibian skin was estimated using the Bayesian SourceTracker (65). SourceTracker was run with the following parameters: rarefaction=1,000 reads, burn-in=1,000, and restart=10.

A phylogenetic null model was used to analyze the forces that drive the micro-eukaryotic community assemblies, using the Beta Nearest Taxon index, a phylogenetic metric that measures how different two microbial communities are in terms of the evolutionary relatedness of their species (66). The Clustal Omega software was used to align the 18S rRNA gene ASV sequences (67). We used the MAFFT software to compute and build the Neighbor-Joining phylogenetic tree (68). The *cophenetic* function from the Stats base R package was used to estimate the phylogenetic distance between sequences. Mean Nearest Taxon Distances (MNTD) were computed with the function *mntd* of the Picante R package version 1.8.2, with the relative abundance of each ASV. MNTD null distribution was computed from random permutations (*n* = 999). βNTI values were computed using the formula:


$$\beta NTI=\frac{MNTD_{obs}-MNTD_{rand}}{SD\left(MNTD_{rand}\right)},$$

with MNTD_obs_ being the average distance to the nearest taxon observed in the community, MNTD_rand_ is the average of the null MNTD distances, and SD(MNTD_rand_) is the standard deviation of null *MNTD* distances. βNTI values near zero show a fully stochastic distribution of communities, whereas values farther from zero indicate the existence of deterministic processes.

## Supplementary Material

Reviewer comments

## Data Availability

The data sets supporting the conclusions of this article are available in NCBI/ENA/DDBJ under the accession number PRJEB91889.

## SUPPLEMENTAL MATERIAL

Supplemental Material msystems.01436-25-s0001.docx Figures S1 to S9; Tables S1 to S12.

OPEN PEER REVIEW reviewer-comments.pdf An accounting of the reviewer comments and feedback.

ASM does not own the copyrights to Supplemental Material that may be linked to, or accessed through, an article. The authors have granted ASM a non-exclusive, world-wide license to publish the Supplemental Material files. Please contact the corresponding author directly for reuse.

## References

[1] Brucker RM, Harris RN, Schwantes CR, Gallaher TN, Flaherty DC, Lam BA, Minbiole KPC. 2008. Amphibian chemical defense: antifungal metabolites of the microsymbiont Janthinobacterium lividum on the salamander Plethodon cinereus. J Chem Ecol 34:1422–1429. doi:10.1007/s10886-008-9555-7 18949519

[2] Hanson MA. 2024. When the microbiome shapes the host: immune evolution implications for infectious disease. Phil Trans R Soc B 379:20230061. doi:10.1098/rstb.2023.0061 38497259 PMC10945400

[3] Honda K, Littman DR. 2012. The microbiome in infectious disease and inflammation. Annu Rev Immunol 30:759–795. doi:10.1146/annurev-immunol-020711-074937 22224764 PMC4426968

[4] Thaiss CA, Zmora N, Levy M, Elinav E. 2016. The microbiome and innate immunity. Nature 535:65–74. doi:10.1038/nature18847 27383981

[5] Woodhams DC, McCartney J, Walke JB, Whetstone R. 2023. The adaptive microbiome hypothesis and immune interactions in amphibian mucus. Dev Comp Immunol 145:104690. doi:10.1016/j.dci.2023.104690 37001710 PMC10249470

[6] Costa L, de Faria MR, Chiaramonte JB, Mendes LW, Sepo E, de Hollander M, Fernandes JMC, Carrión VJ, Bettiol W, Mauchline TH, Raaijmakers JM, Mendes R. 2023. Repeated exposure of wheat to the fungal root pathogen Bipolaris sorokiniana modulates rhizosphere microbiome assembly and disease suppressiveness. Environ Microbiome 18:85. doi:10.1186/s40793-023-00529-2 38053159 PMC10696838

[7] Parajuli A, Grönroos M, Siter N, Puhakka R, Vari HK, Roslund MI, Jumpponen A, Nurminen N, Laitinen OH, Hyöty H, Rajaniemi J, Sinkkonen A. 2018. Urbanization reduces transfer of diverse environmental microbiota indoors. Front Microbiol 9:84. doi:10.3389/fmicb.2018.00084 29467728 PMC5808279

[8] Loyau A, Bouchali R, Sentenac H, Schmeller DS. 2024. The commensal skin microbiome of amphibian mountain populations and its association with the pathogen Batrachochytrium dendrobatidis. Environ Microbiol 26:e16699. doi:10.1111/1462-2920.16699 39374928

[9] Rebollar EA, Simonetti SJ, Shoemaker WR, Harris RN. 2016. Direct and indirect horizontal transmission of the antifungal probiotic bacterium Janthinobacterium lividum on green frog (Lithobates clamitans) Tadpoles. Appl Environ Microbiol 82:2457–2466. doi:10.1128/AEM.04147-15 26873311 PMC4959476

[10] Leveau JHJ. 2024. Re-envisioning the plant disease triangle: full integration of the host microbiota and a focal pivot to health outcomes. Annu Rev Phytopathol 62:31–47. doi:10.1146/annurev-phyto-121423-042021 38684078

[11] Bernardo-Cravo AP, Schmeller DS, Chatzinotas A, Vredenburg VT, Loyau A. 2020. Environmental factors and host microbiomes shape host-pathogen dynamics. Trends Parasitol 36:616–633. doi:10.1016/j.pt.2020.04.010 32402837

[12] Ma Z, Zhang Y-P. 2022. Ecology of human medical enterprises: from disease ecology of zoonoses, cancer ecology through to medical ecology of human microbiomes. Front Ecol Evol 10:879130. doi:10.3389/fevo.2022.879130

[13] Slippers B. 2020. The plant disease pyramid: the relevance of the original vision of plant pathology in 2020. S Afr J Sci 116. doi:10.17159/sajs.2020/9011

[14] Sentenac H, Schmeller DS, Caubet S, Carsin A, Guillet R, Ferriol J, Leflaive J, Loyau A. 2024. Biofilms inactivate the free-living stage of Batrachochytrium dendrobatidis, the most destructive pathogen for vertebrate diversity. ISME J 18:wrae189. doi:10.1093/ismejo/wrae189 39325976 PMC11630259

[15] Schlatter D, Kinkel L, Thomashow L, Weller D, Paulitz T. 2017. Disease suppressive soils: new insights from the soil microbiome. Phytopathology 107:1284–1297. doi:10.1094/PHYTO-03-17-0111-RVW 28650266

[16] Schmeller DS, Blooi M, Martel A, Garner TWJ, Fisher MC, Azemar F, Clare FC, Leclerc C, Jäger L, Guevara-Nieto M, Loyau A, Pasmans F. 2014. Microscopic aquatic predators strongly affect infection dynamics of a globally emerged pathogen. Curr Biol 24:176–180. doi:10.1016/j.cub.2013.11.032 24374305

[17] Longcore JE, Pessier AP, Nichols DK. 1999. Batrachochytrium dendrobatidis gen. et sp. nov., a chytrid pathogenic to amphibians. Mycologia 91:219–227. doi:10.1080/00275514.1999.12061011

[18] Scheele BC, Pasmans F, Skerratt LF, Berger L, Martel A, Beukema W, Acevedo AA, Burrowes PA, Carvalho T, Catenazzi A, et al.. 2019. Amphibian fungal panzootic causes catastrophic and ongoing loss of biodiversity. Science 363:1459–1463. doi:10.1126/science.aav0379 30923224

[19] Alexiev A, Chen MY, Korpita T, Weier AM, McKenzie VJ. 2023. Together or alone: evaluating the pathogen inhibition potential of bacterial cocktails against an amphibian pathogen. Microbiol Spectr 11:e0151822. doi:10.1128/spectrum.01518-22 36719234 PMC10100949

[20] Kueneman JG, Parfrey LW, Woodhams DC, Archer HM, Knight R, McKenzie VJ. 2014. The amphibian skin-associated microbiome across species, space and life history stages. Mol Ecol 23:1238–1250. doi:10.1111/mec.12510 24171949

[21] Walke JB, Becker MH, Loftus SC, House LL, Cormier G, Jensen RV, Belden LK. 2014. Amphibian skin may select for rare environmental microbes. ISME J 8:2207–2217. doi:10.1038/ismej.2014.77 24858782 PMC4992085

[22] Keeling PJ, Campo J del. 2017. Marine protists are not just big bacteria. Curr Biol 27:R541–R549. doi:10.1016/j.cub.2017.03.075 28586691

[23] Vargas-Albores F, Garibay-Valdez E, Medina-Félix D, Martínez-Porchas M. 2023. The micro-eukaryotic community: an underrated component of the mammalian gut microbiota? Front Microbiol 14:1123513. doi:10.3389/fmicb.2023.1123513 37007497 PMC10060968

[24] Laforest-Lapointe I, Arrieta M-C. 2018. Microbial eukaryotes: a missing link in gut microbiome studies. mSystems 3:e00201-17. doi:10.1128/mSystems.00201-17 29556538 PMC5850078

[25] Heck N, Freudenthal J, Dumack K. 2023. Microeukaryotic predators shape the wastewater microbiome. Water Res 242:120293. doi:10.1016/j.watres.2023.120293 37421865

[26] Chudnovskiy A, Mortha A, Kana V, Kennard A, Ramirez JD, Rahman A, Remark R, Mogno I, Ng R, Gnjatic S, Amir E-AD, Solovyov A, Greenbaum B, Clemente J, Faith J, Belkaid Y, Grigg ME, Merad M. 2016. Host-protozoan interactions protect from mucosal infections through activation of the inflammasome. Cell 167:444–456. doi:10.1016/j.cell.2016.08.076 27716507 PMC5129837

[27] del Campo J, Bass D, Keeling PJ. 2020. The eukaryome: diversity and role of microeukaryotic organisms associated with animal hosts. Funct Ecol 34:2045–2054. doi:10.1111/1365-2435.13490

[28] Rebollar EA, Martínez-Ugalde E, Orta AH. 2020. The amphibian skin microbiome and its protective role against chytridiomycosis. Herpetologica 76:167. doi:10.1655/0018-0831-76.2.167

[29] Kearns PJ, Fischer S, Fernández-Beaskoetxea S, Gabor CR, Bosch J, Bowen JL, Tlusty MF, Woodhams DC. 2017. Fight fungi with fungi: antifungal properties of the amphibian mycobiome. Front Microbiol 8:2494. doi:10.3389/fmicb.2017.02494 29312201 PMC5735112

[30] Alexiev A, Melie T, Martindale R, Delacey C, Quandt CA, McKenzie VJ. 2023. Mr. Toad’s wild fungi: fungal isolate diversity on colorado boreal toads and their capacity for pathogen inhibition. Fungal Ecol 66:101297. doi:10.1016/j.funeco.2023.101297 38487623 PMC10938945

[31] Medina D, Hughey MC, Walke JB, Becker MH, Pontarelli K, Sun S, Badgley B, Belden LK. 2019. Amphibian skin fungal communities vary across host species and do not correlate with infection by a pathogenic fungus. Environ Microbiol 21:2905–2920. doi:10.1111/1462-2920.14682 31087743

[32] Altig R, Whiles MR, Taylor CL. 2007. What do tadpoles really eat? Assessing the trophic status of an understudied and imperiled group of consumers in freshwater habitats. Freshw Biol 52:386–395. doi:10.1111/j.1365-2427.2006.01694.x

[33] Sentenac H, Loyau A, Zoccarato L, Jassey VEJ, Grossart H-P, Schmeller DS. 2023. Biofilm community composition is changing in remote mountain lakes with a relative increase in potentially toxigenic algae. Water Res 245:120547. doi:10.1016/j.watres.2023.120547 37708771

[34] Sentenac H, Loyau A, Leflaive J, Schmeller DS. 2022. The significance of biofilms to human, animal, plant and ecosystem health. Funct Ecol 36:294–313. doi:10.1111/1365-2435.13947

[35] Elton CS. 1958. The ecology of invasions by animals and plants. Methuen, London.

[36] Xun W, Liu Y, Li W, Ren Y, Xiong W, Xu Z, Zhang N, Miao Y, Shen Q, Zhang R. 2021. Specialized metabolic functions of keystone taxa sustain soil microbiome stability. Microbiome 9:35. doi:10.1186/s40168-020-00985-9 33517892 PMC7849160

[37] van Elsas JD, Chiurazzi M, Mallon CA, Elhottova D, Kristufek V, Salles JF. 2012. Microbial diversity determines the invasion of soil by a bacterial pathogen. Proc Natl Acad Sci USA 109:1159–1164. doi:10.1073/pnas.1109326109 22232669 PMC3268289

[38] Devevey G, Dang T, Graves CJ, Murray S, Brisson D. 2015. First arrived takes all: inhibitory priority effects dominate competition between co-infecting Borrelia burgdorferi strains. BMC Microbiol 15:61. doi:10.1186/s12866-015-0381-0 25887119 PMC4359528

[39] Keesing F, Belden LK, Daszak P, Dobson A, Harvell CD, Holt RD, Hudson P, Jolles A, Jones KE, Mitchell CE, Myers SS, Bogich T, Ostfeld RS. 2010. Impacts of biodiversity on the emergence and transmission of infectious diseases. Nature 468:647–652. doi:10.1038/nature09575 21124449 PMC7094913

[40] Kinnunen M, Dechesne A, Proctor C, Hammes F, Johnson D, Quintela-Baluja M, Graham D, Daffonchio D, Fodelianakis S, Hahn N, Boon N, Smets BF. 2016. A conceptual framework for invasion in microbial communities. ISME J 10:2773–2775. doi:10.1038/ismej.2016.75 27137125 PMC5148196

[41] Debray R, Herbert RA, Jaffe AL, Crits-Christoph A, Power ME, Koskella B. 2022. Priority effects in microbiome assembly. Nat Rev Microbiol 20:109–121. doi:10.1038/s41579-021-00604-w 34453137

[42] Patsch D, van Vliet S, Marcantini LG, Johnson DR. 2018. Generality of associations between biological richness and the rates of metabolic processes across microbial communities. Environ Microbiol 20:4356–4368. doi:10.1111/1462-2920.14352 29984466

[43] Wickham SA, Claessens M, Post AF. 2015. Ciliates, microbes and nutrients: interactions in the seasonally mixed Gulf of Aqaba. J Plankton Res 37:258–271. doi:10.1093/plankt/fbu103

[44] Kagami M, Miki T, Takimoto G. 2014. Mycoloop: chytrids in aquatic food webs. Front Microbiol 5:166. doi:10.3389/fmicb.2014.00166 24795703 PMC4001071

[45] Jani AJ, Briggs CJ. 2014. The pathogen Batrachochytrium dendrobatidis disturbs the frog skin microbiome during a natural epidemic and experimental infection. Proc Natl Acad Sci USA 111:E5049–E5058. doi:10.1073/pnas.1412752111 25385615 PMC4250152

[46] Grossart H-P, Van den Wyngaert S, Kagami M, Wurzbacher C, Cunliffe M, Rojas-Jimenez K. 2019. Fungi in aquatic ecosystems. Nat Rev Microbiol 17:339–354. doi:10.1038/s41579-019-0175-8 30872817

[47] Zhang X, Dong H, Zheng P, Li G, He C, Guo X, Zhang J, Gong J. 2023. The habitat differentiation, dynamics and functional potentials of bacterial and micro-eukaryotic communities in shrimp aquaculture systems with limited water exchange. Aquaculture 566:739156. doi:10.1016/j.aquaculture.2022.739156

[48] Ng MS, Soon N, Chin MY, Ho SK, Drescher L, Sani MAB, Lim KE, Wainwright BJ, Chang Y. 2025. Fungi promote cross-domain interactions even in deep anoxic mangrove sediments. Environ Microbiome 20:34. doi:10.1186/s40793-025-00686-6 40133912 PMC11934577

[49] Belasen AM, Riolo MA, Bletz MC, Lyra ML, Toledo LF, James TY. 2021. Geography, host genetics, and cross-domain microbial networks structure the skin microbiota of fragmented Brazilian Atlantic forest frog populations. Ecol Evol 11:9293–9307. doi:10.1002/ece3.7594 34306622 PMC8293785

[50] Garner TWJ, Walker S, Bosch J, Leech S, Marcus Rowcliffe J, Cunningham AA, Fisher MC. 2009. Life history tradeoffs influence mortality associated with the amphibian pathogen Batrachochytrium dendrobatidis. Oikos 118:783–791. doi:10.1111/j.1600-0706.2008.17202.x

[51] Bosch J, Martínez-Solano I, García-París M. 2001. Evidence of a chytrid fungus infection involved in the decline of the common midwife toad (Alytes obstetricans) in protected areas of central Spain. Biol Conserv 97:331–337. doi:10.1016/S0006-3207(00)00132-4

[52] Davis LR, Bigler L, Woodhams DC. 2017. Developmental trajectories of amphibian microbiota: response to bacterial therapy depends on initial community structure. Environ Microbiol 19:1502–1517. doi:10.1111/1462-2920.13707 28229543

[53] Marantelli G, Berger L, Speare R, Keegan L. 2004. Distribution of the amphibian chytrid Batrachochytrium dendrobatidis and keratin during tadpole development. Pacific Conserv Biol 10:173–179. doi:10.1071/PC040173

[54] McMahon TA, Rohr JR. 2015. Transition of chytrid fungus infection from mouthparts to hind limbs during amphibian metamorphosis. Ecohealth 12:188–193. doi:10.1007/s10393-014-0989-9 25384612

[55] Amaral-Zettler LA, McCliment EA, Ducklow HW, Huse SM. 2009. A method for studying protistan diversity using massively parallel sequencing of V9 hypervariable regions of small-subunit ribosomal RNA genes. PLoS One 4:e6372. doi:10.1371/journal.pone.0006372 19633714 PMC2711349

[56] Bradley IM, Pinto AJ, Guest JS. 2016. Design and evaluation of Illumina MiSeq-compatible, 18S rRNA gene-specific primers for improved characterization of mixed phototrophic communities. Appl Environ Microbiol 82:5878–5891. doi:10.1128/AEM.01630-16 27451454 PMC5038042

[57] Martin M. 2011. Cutadapt removes adapter sequences from high-throughput sequencing reads. EMBnet J 17:10. doi:10.14806/ej.17.1.200

[58] Callahan BJ, McMurdie PJ, Rosen MJ, Han AW, Johnson AJA, Holmes SP. 2016. DADA2: high-resolution sample inference from Illumina amplicon data. Nat Methods 13:581–583. doi:10.1038/nmeth.3869 27214047 PMC4927377

[59] Wang Q, Garrity GM, Tiedje JM, Cole JR. 2007. Naive Bayesian classifier for rapid assignment of rRNA sequences into the new bacterial taxonomy. Appl Environ Microbiol 73:5261–5267. doi:10.1128/AEM.00062-07 17586664 PMC1950982

[60] Davis NM, Proctor DM, Holmes SP, Relman DA, Callahan BJ. 2018. Simple statistical identification and removal of contaminant sequences in marker-gene and metagenomics data. Microbiome 6:226. doi:10.1186/s40168-018-0605-2 30558668 PMC6298009

[61] Dixon P. 2003. VEGAN, a package of R functions for community ecology. J Vegetation Science 14:927–930. doi:10.1111/j.1654-1103.2003.tb02228.x

[62] Wickham H. 2016. ggplot2. Springer International Publishing, Cham. http://link.springer.com/10.1007/978-3-319-24277-4.

[63] Farthing HN, Jiang J, Henwood AJ, Fenton A, Garner TWJ, Daversa DR, Fisher MC, Montagnes DJS. 2020. Microbial grazers may aid in controlling infections caused by the aquatic zoosporic fungus Batrachochytrium dendrobatidis. Front Microbiol 11:592286. doi:10.3389/fmicb.2020.592286 33552011 PMC7858660

[64] Lin H, Peddada SD. 2020. Analysis of compositions of microbiomes with bias correction. Nat Commun 11:3514. doi:10.1038/s41467-020-17041-7 32665548 PMC7360769

[65] Knights D, Kuczynski J, Charlson ES, Zaneveld J, Mozer MC, Collman RG, Bushman FD, Knight R, Kelley ST. 2011. Bayesian community-wide culture-independent microbial source tracking. Nat Methods 8:761–763. doi:10.1038/nmeth.1650 21765408 PMC3791591

[66] Custer GF, Bresciani L, Dini-Andreote F. 2024. Toward an integrative framework for microbial community coalescence. Trends Microbiol 32:241–251. doi:10.1016/j.tim.2023.09.001 37778924

[67] Sievers F, Wilm A, Dineen D, Gibson TJ, Karplus K, Li W, Lopez R, McWilliam H, Remmert M, Söding J, Thompson JD, Higgins DG. 2011. Fast, scalable generation of high-quality protein multiple sequence alignments using Clustal Omega. Mol Syst Biol 7:539. doi:10.1038/msb.2011.75 21988835 PMC3261699

[68] Katoh K, Misawa K, Kuma K, Miyata T. 2002. MAFFT: a novel method for rapid multiple sequence alignment based on fast Fourier transform. Nucleic Acids Res 30:3059–3066. doi:10.1093/nar/gkf436 12136088 PMC135756

