# Supplementary material for: Unraveling the disease pyramid: the role of environmental micro-eukaryotes in amphibian resistance to the deadly fungal pathogen Batrachochytrium dendrobatidis
Source: mSystems. 2025 Dec 15;11(1):e01436-25. doi: 10.1128/msystems.01436-25 (PMC12817952; doi:10.1128/msystems.01436-25)
Supplement: Supplemental Material — Figures S1 to S9; Tables S1 to S12. [file msystems.01436-25-s0001.docx]

**
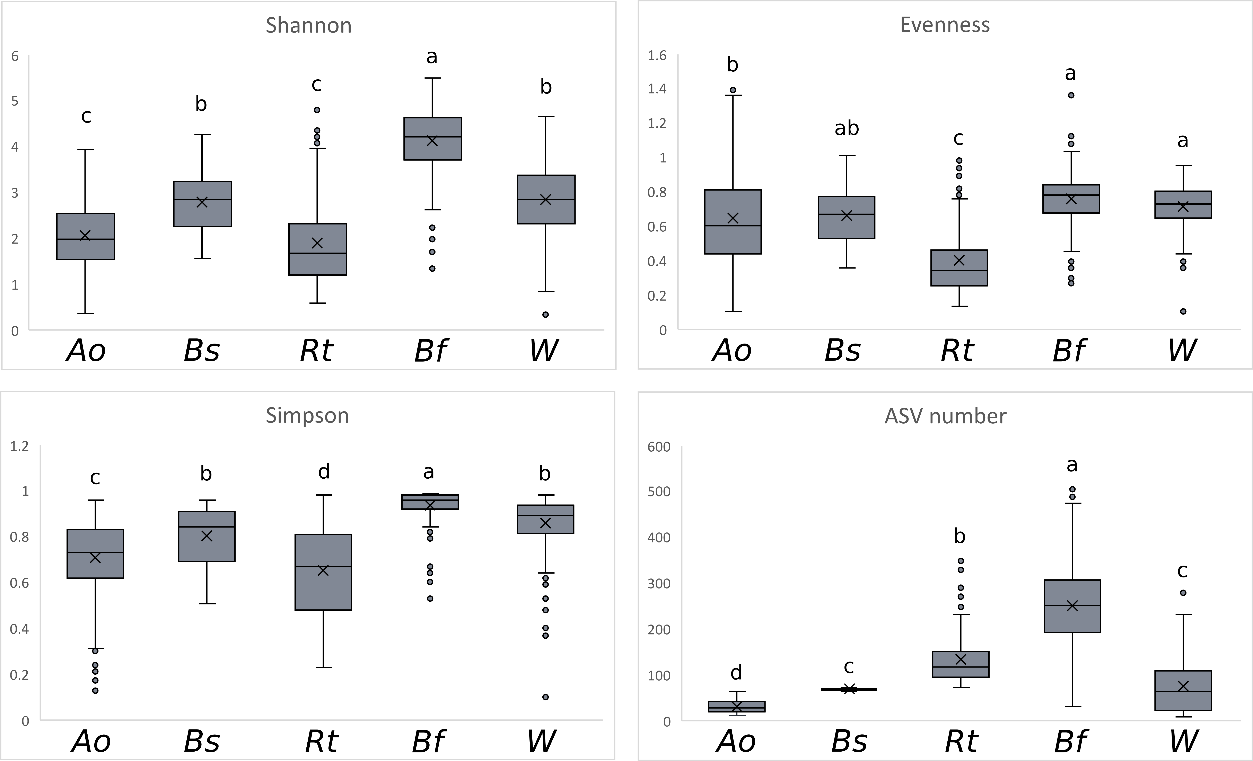
**

**Fig. S1.**

Diversity indexes computed from amphibian tadpole skin, biofilm and water samples. The different letters indicate groups significantly differentiated from each other’s (ANOVA test followed by Tukey post-hoc test, p<0.05). On each bar, the cross represents the mean, while the line shows the median. *Ao* = *A. obstetricans,* *Bs* = *B. spinosus*, *Rt* = *R. temporaria*, Bf = Biofilm and W = Water.

**
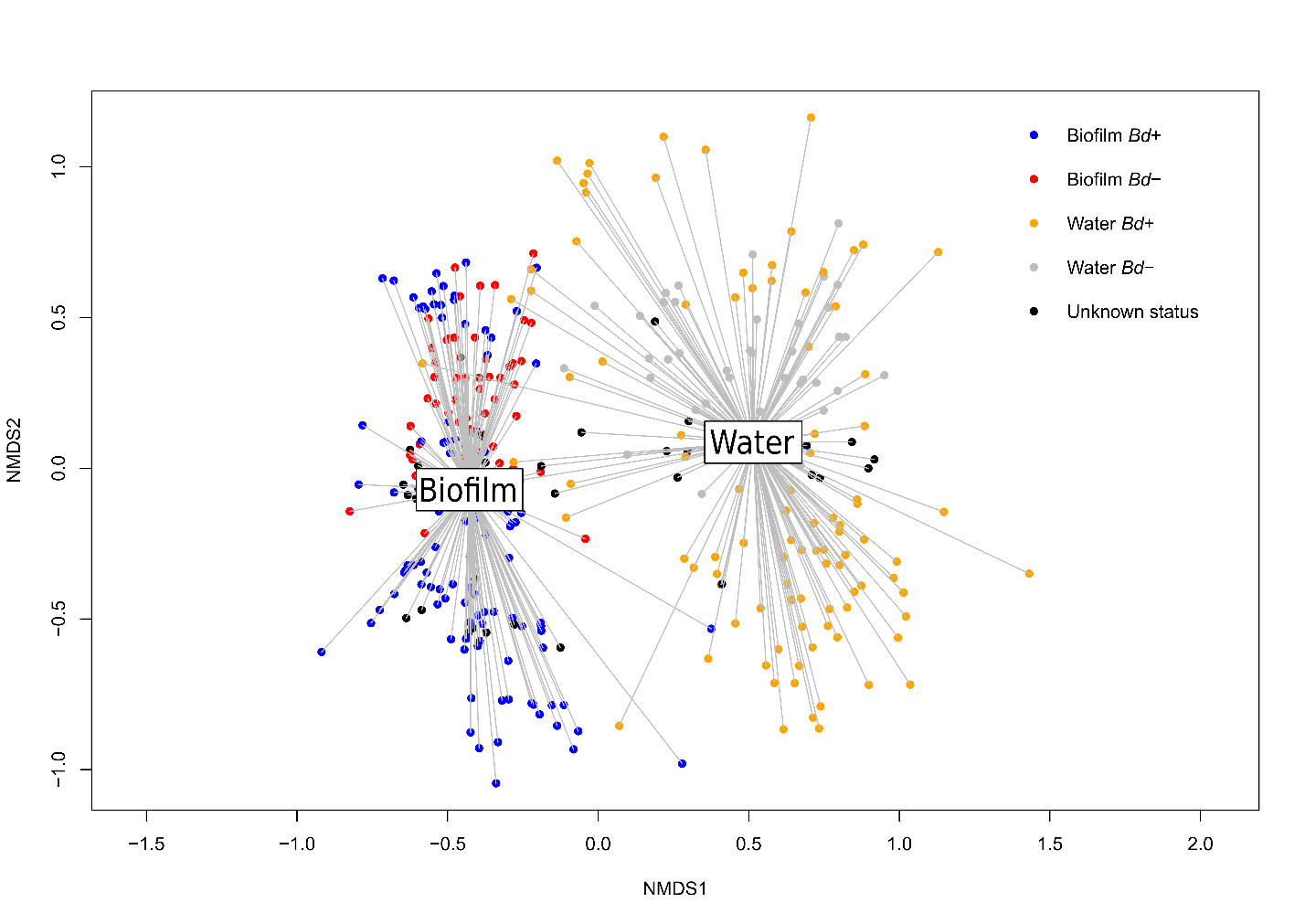
Fig. S2.**

NMDS ordination based on Bray-Curtis dissimilarities computed from biofilm and water samples. Samples from the same habitat (water or biofilm) were grouped using the *ordispider* function of the vegan R package. Colors indicate *Bd* infection status. Stress = 0.16.


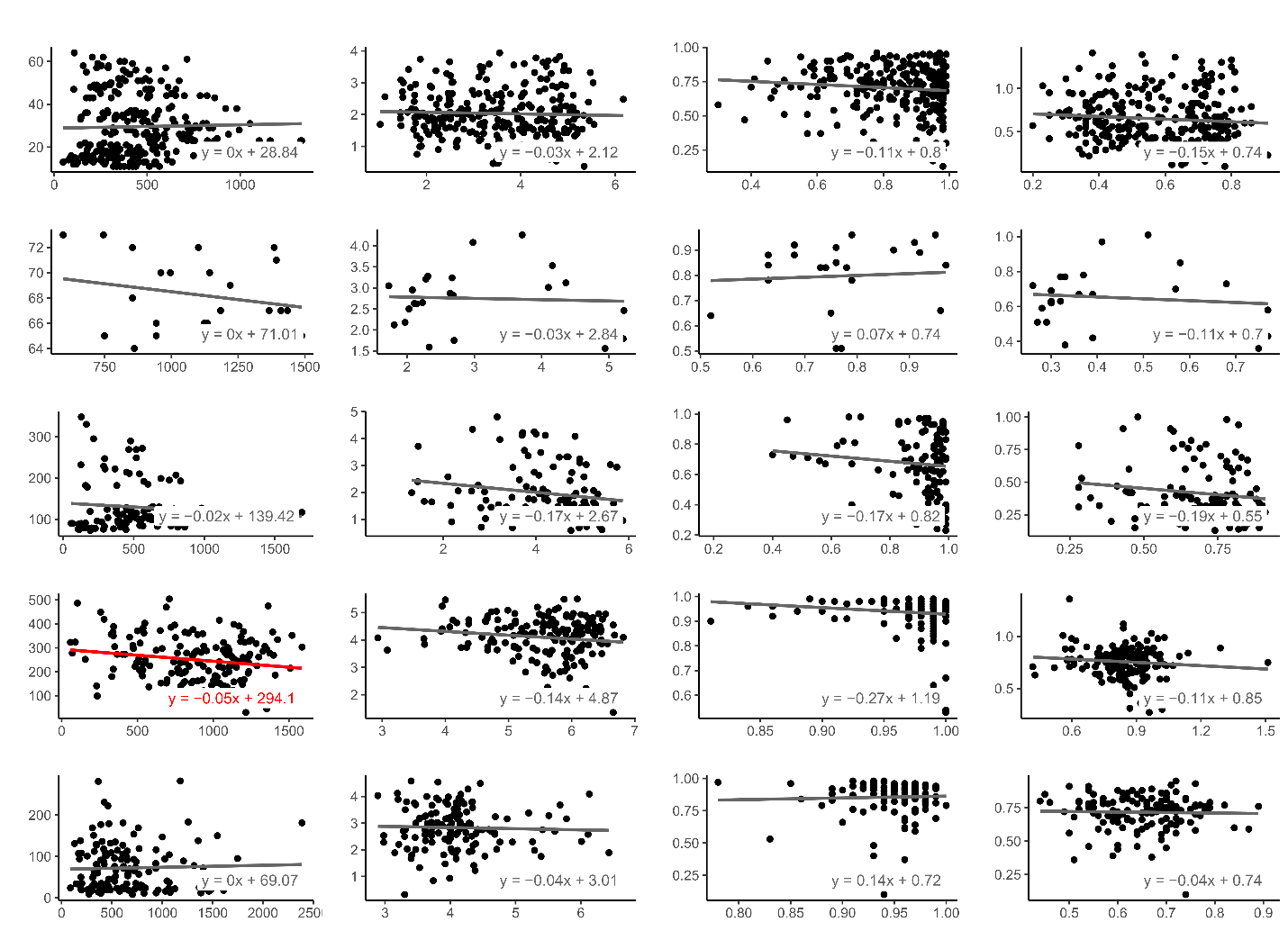


ASV number

Shannon

Simpson

Evenness

Ao

*Bs*

*Rt*

W

Bf

**Fig. S3.** Linear regression showing the relationship between α-diversity indexes computed from the 16S (bacterial communities, x axis) and 18S (micro-eukaryotic communities, y axis) ASVs. The regression line colored in red shows a significant relationship.


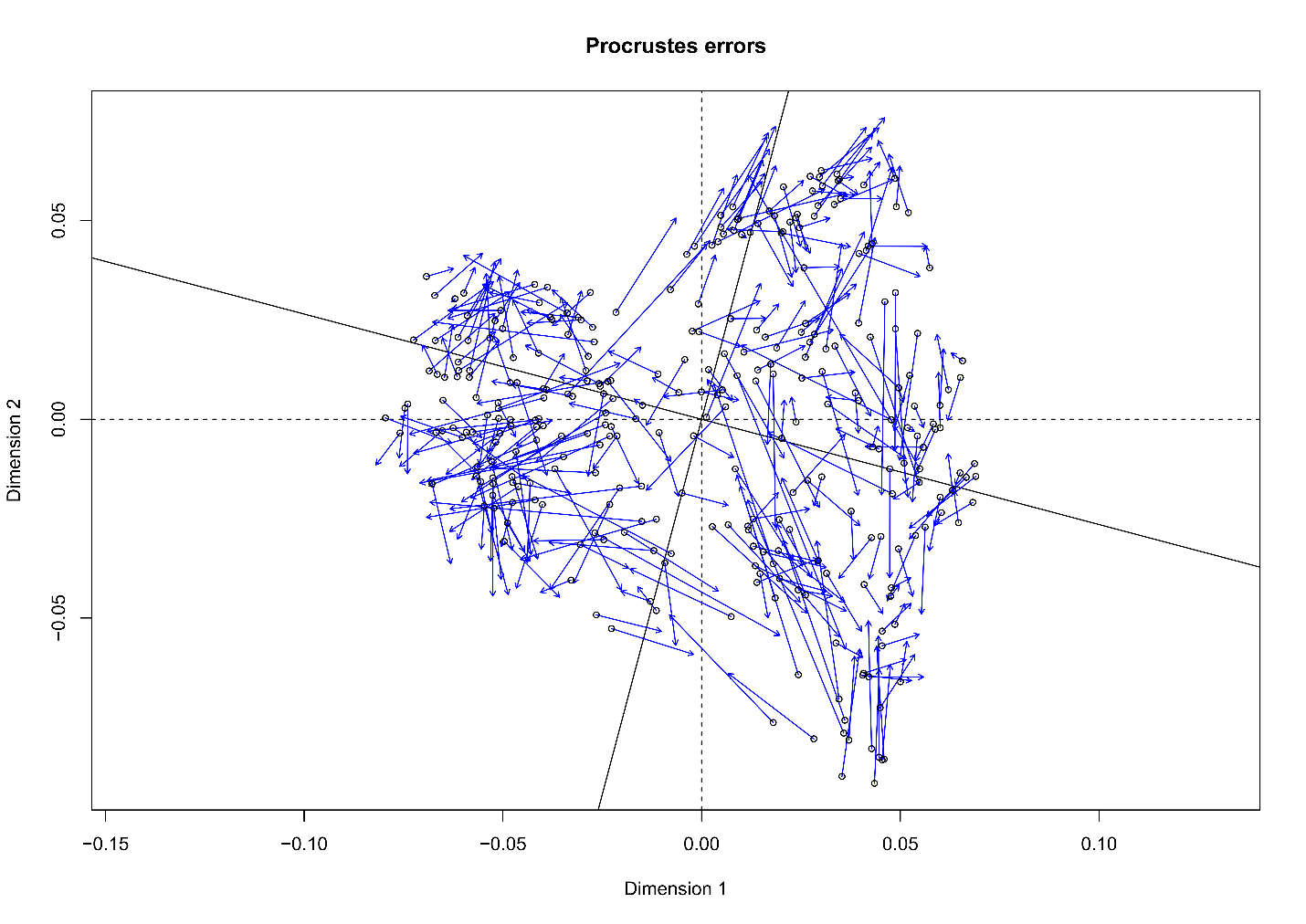


**Fig. S4A**. Procrustes errors plots illustrating the congruence between the 16S (bacterial communities) and 18S (micro-eukaryotic communities) ASVs NMDS ordination (Bray-Curtis distances) from water and biofilm samples. Arrows indicate the direction and magnitude of residual differences after Procrustes superposition, with shorter arrows indicating better concordance.


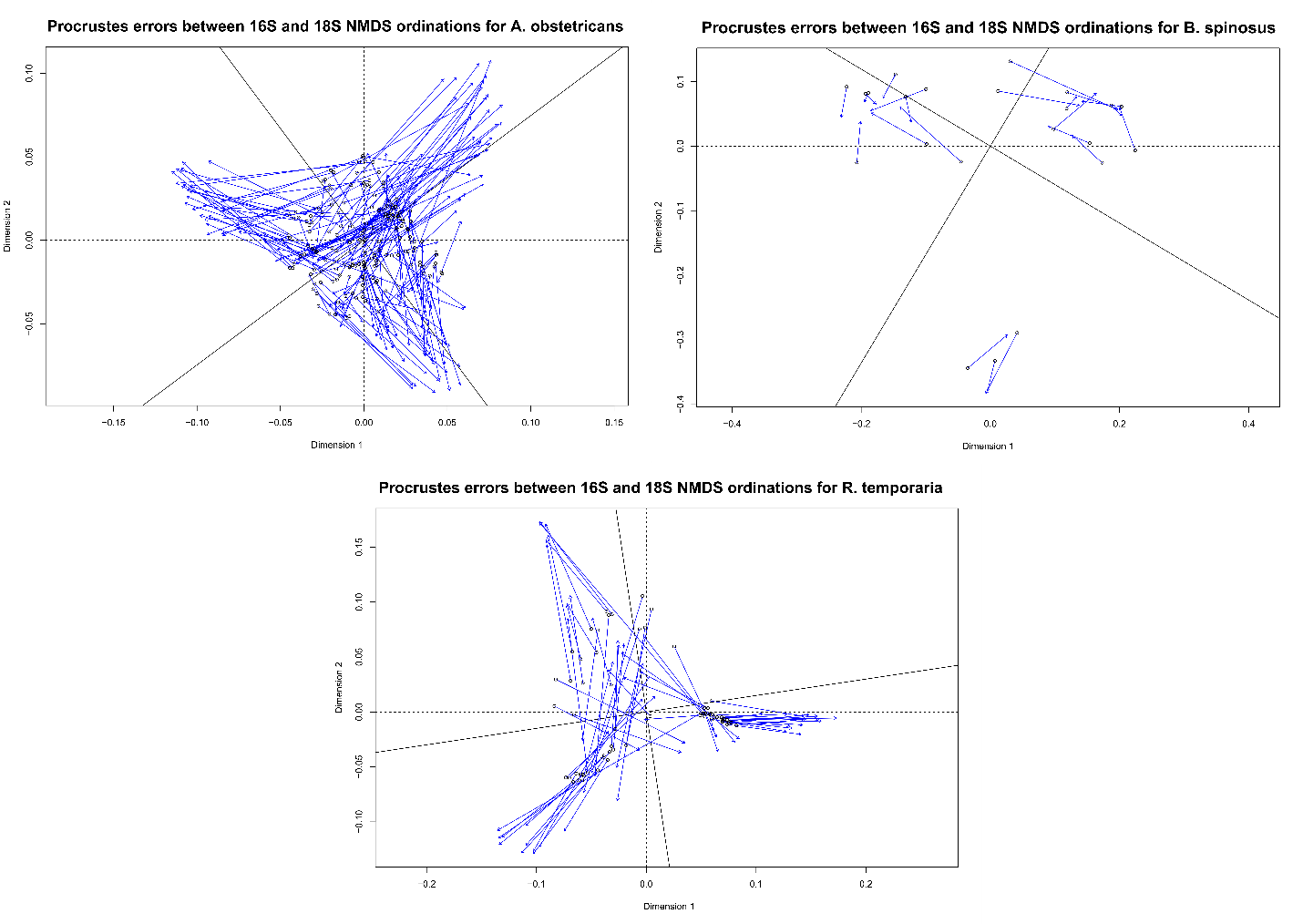


**Fig. S4b**

**Fig. S4B.** Procrustes errors plots illustrating the congruence between the 16S (bacterial communities) and 18S (micro-eukaryotic communities) ASVs NMDS ordination (Bray-Curtis distances) from A. obstetricans, B. spinosus and R. temporaria skin samples. Arrows indicate the direction and magnitude of residual differences after Procrustes superposition, with shorter arrows indicating better concordance.


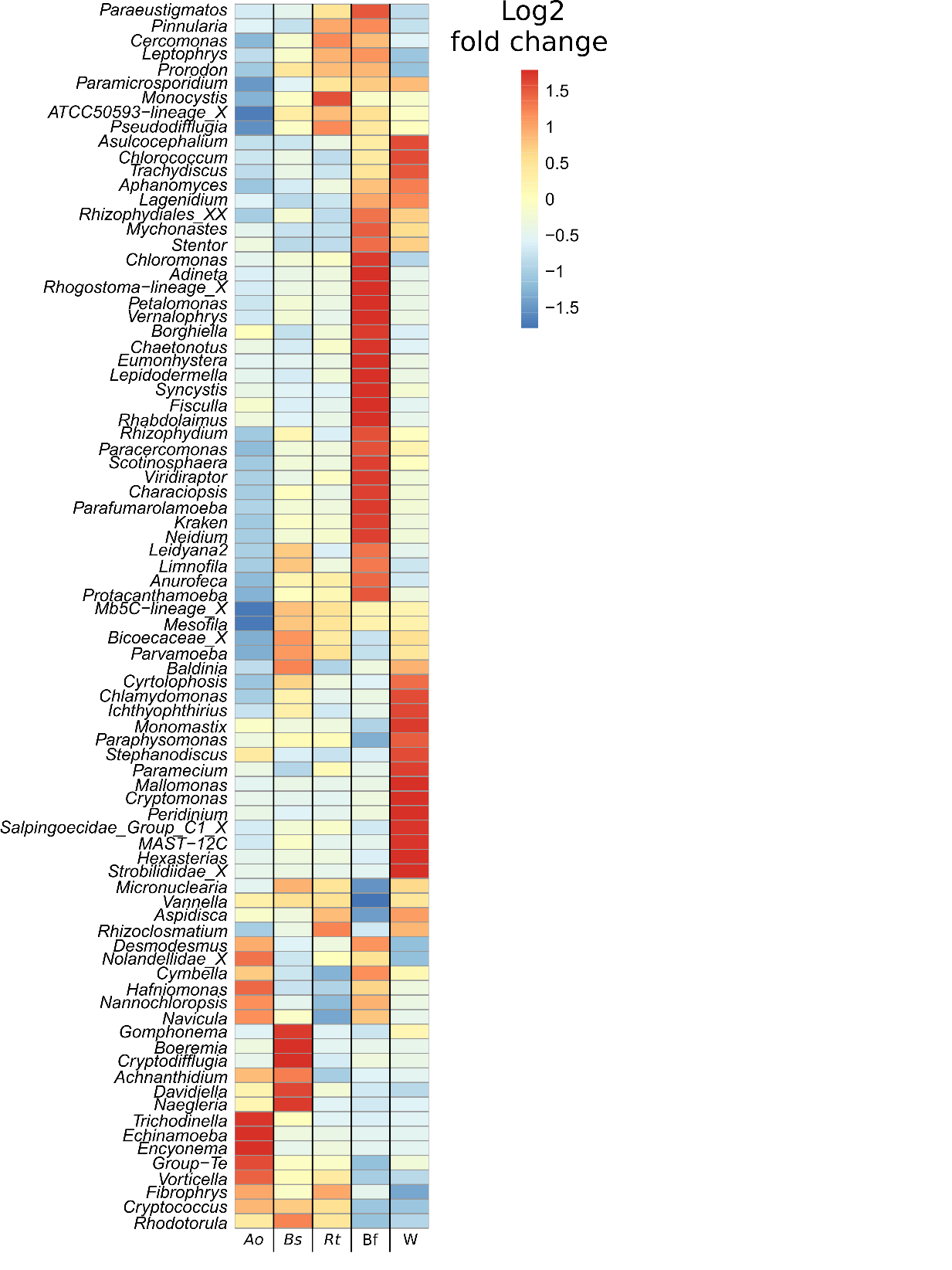


**Fig. S5.** Heatmap of the micro-eukaryotic genera differently associated to the host species, the biofilm and the water samples, as revealed by analysis of compositions of microbiomes with bias correction (ANCOM-BC). When the genus was unknown, the family (_X) or the order (_XX) is indicated. Only taxa with a significant overall distribution bias are shown. Ao = A. obstetricans, Bs = B. spinosus, Rt = R. temporaria, Bf = Biofilm and W = Water.


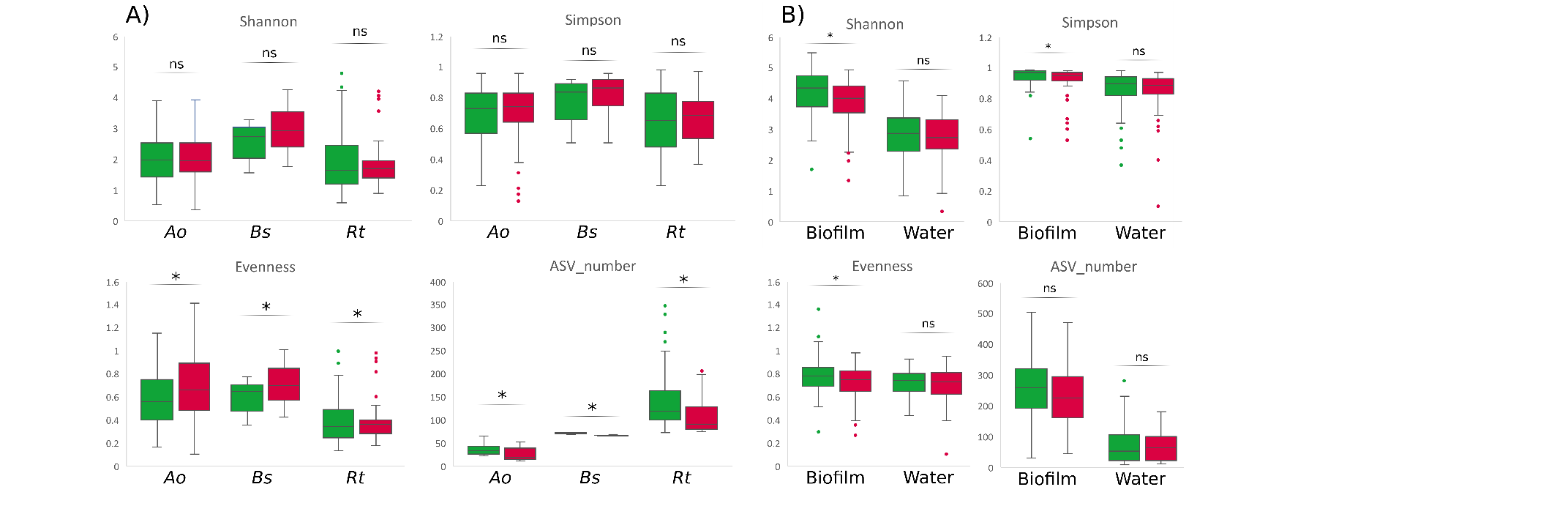


**Fig. S6.** Diversity indexes computed from A) skin samples of each amphibian host and B) environmental biofilm and water samples, both according to the Bd infection status of the lakes (green: Bd-negative, red: Bd-positive). Asterisks highlight significant differences based on the Wilcoxon test (p<0.05). Ao = A. obstetricans, Bs = B. spinosus and Rt = R. temporaria.

**
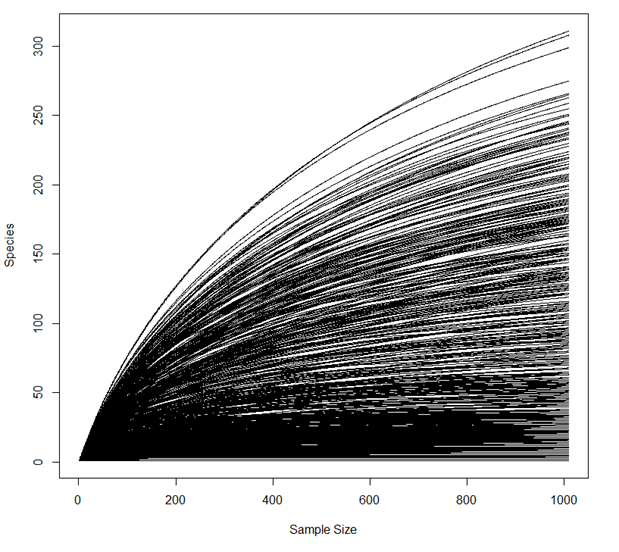
**

**Fig. S7.** Rarefaction curves of the 18S rRNA gene rarefied cleaned reads.


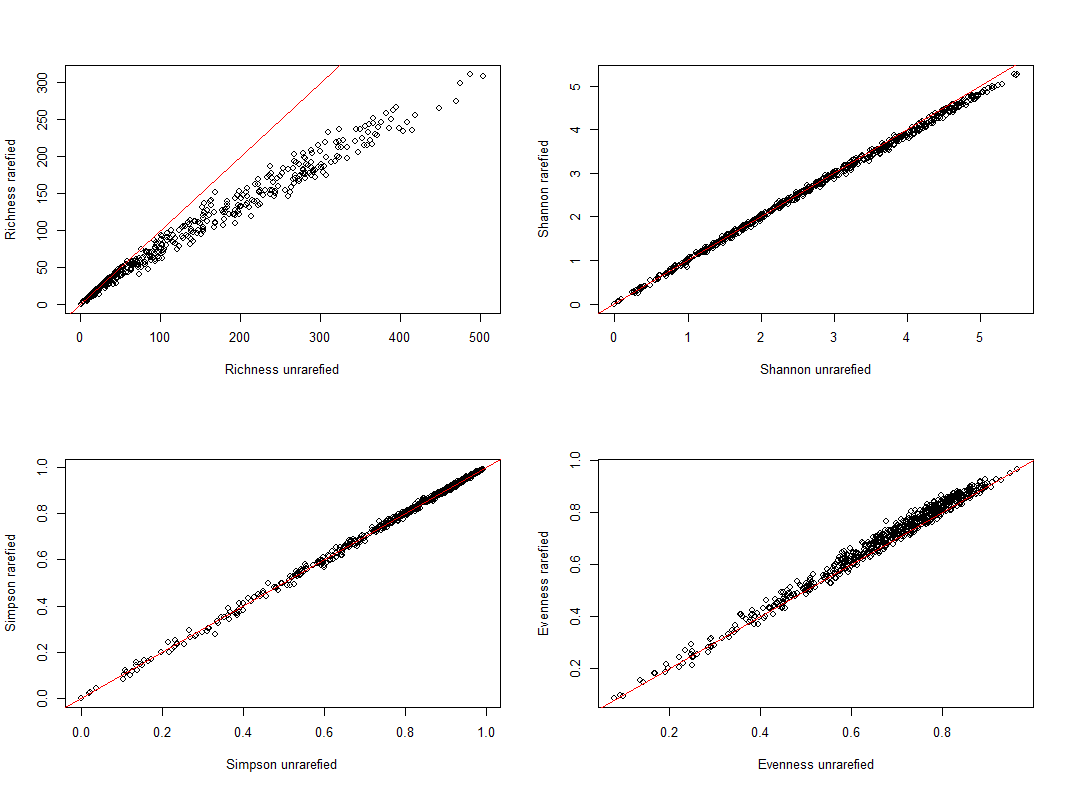


**Fig. S8.** Scatter plots showing the correlation between the richness (ASV number) and diversity indexes (Shannon, Simpson and Evenness) computed from the 18S rRNA gene unrarefied (x axis) and rarefied (y axis) dataset.


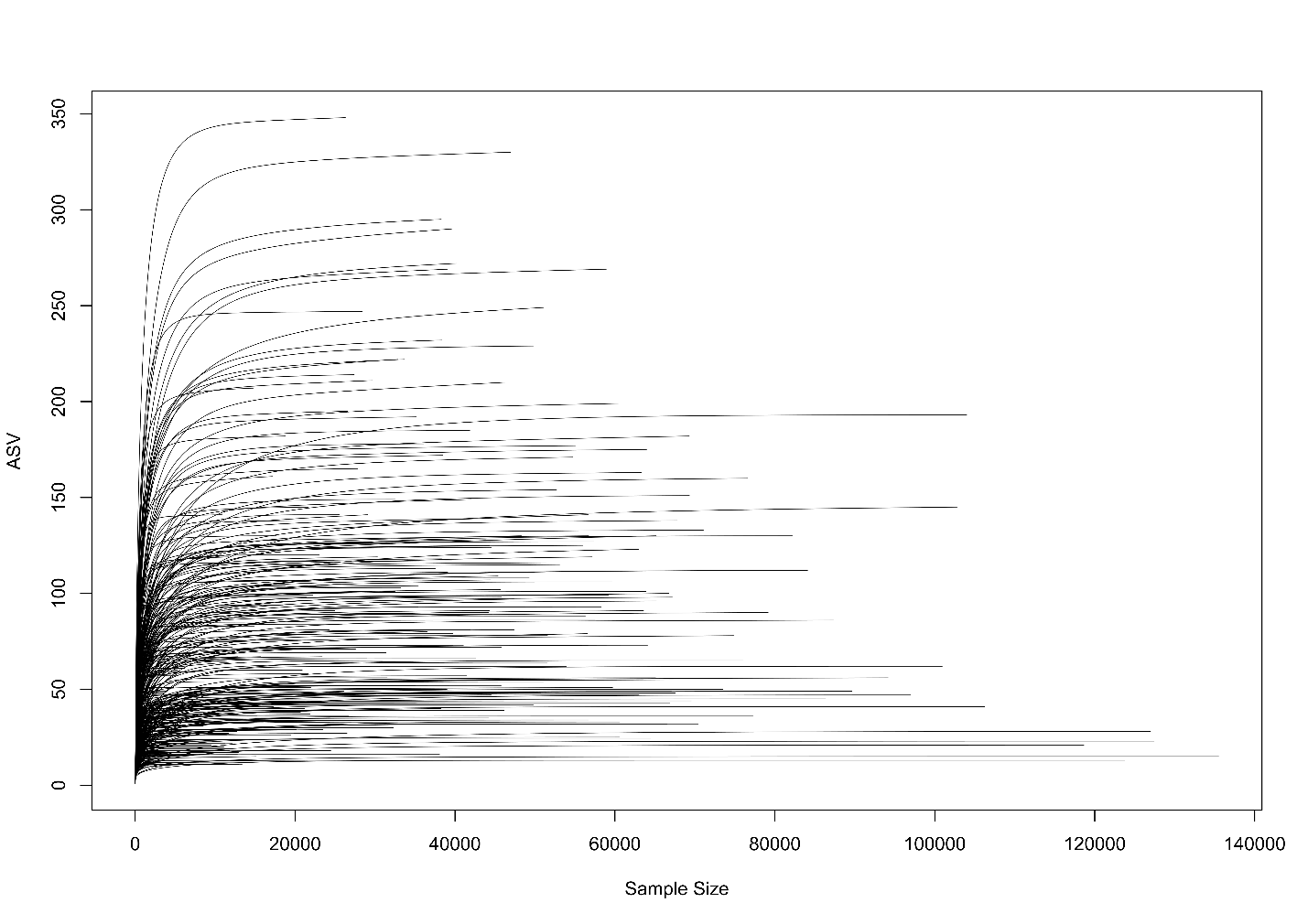


**Fig. S9.** Rarefaction curves of the 18S rRNA gene unrarefied cleaned reads.

**Table S1.** Richness and diversity of the amphibian tadpole skin, the biofilm and the water samples. The average value, its standard deviation, as well as the minimal and maximal value for each index are indicated. Grey cases showed significant difference between the values computed from Bd-negative and Bd-positive lakes.

| ***Bd status*** | **Compartment** | **ASV number** | | | | **Shannon** | | | | **Simpson** | | | | ***Evenness*** | | | |
| --- | --- | --- | --- | --- | --- | --- | --- | --- | --- | --- | --- | --- | --- | --- | --- | --- | --- |
|  |  | Mean | Sd | Min | Max | Mean | Sd | Min | Max | Mean | Sd | Min | Max | Mean | Sd | Min | Max |
| All | *A. obstetricans* | 29.53 | 14.31 | 11 | 64 | 2.03 | 0.76 | 0.37 | 3.94 | 0.70 | 0.18 | 0.13 | 0.96 | 0.65 | 0.28 | 0.1 | 1.00 |
|  | *B. spinosus* | 68.30 | 2.79 | 64 | 73 | 2.79 | 0.71 | 1.56 | 4.26 | 0.80 | 0.16 | 0.51 | 0.96 | 0.66 | 0.17 | 0.36 | 1.00 |
|  | *R. temporaria* | 129.95 | 60.68 | 73 | 348 | 1.98 | 0.98 | 0.59 | 4.80 | 0.67 | 0.20 | 0.23 | 0.98 | 0.42 | 0.21 | 0.13 | 1.00 |
|  | Biofilm | 249.81 | 93.99 | 30 | 504 | 4.09 | 0.74 | 1.35 | 5.50 | 0.93 | 0.08 | 0.53 | 0.99 | 0.76 | 0.15 | 0.27 | 1.00 |
|  | Water | 72.12 | 56.86 | 8 | 282 | 2.83 | 0.79 | 0.32 | 4.58 | 0.86 | 0.13 | 0.10 | 0.98 | 0.72 | 0.13 | 0.10 | 0.95 |
| *Bd-*negative | *A. obstetricans* | **35.76** | 13.42 | 22 | 64 | 1.99 | 0.80 | 0.54 | 3.72 | 0.68 | 0.19 | 0.23 | 0.96 | **0.58** | 0.26 | 0.16 | 1.0 |
|  | *B. spinosus* | **71.20** | 1.33 | 69 | 73 | 2.56 | 0.58 | 1.56 | 3.28 | 0.79 | 0.13 | 0.51 | 0.92 | **0.60** | 0.14 | 0.36 | 0.77 |
|  | *R. temporaria* | **139.33** | 66.42 | 75 | 348 | 2.03 | 1.03 | 0.59 | 4.80 | 0.67 | 0.21 | 0.23 | 0.98 | **0.42** | 0.21 | 0.13 | 1.00 |
|  | Biofilm | 256.87 | 94.04 | 30 | 504 | **4.20** | 0.74 | 1.70 | 5.50 | **0.94** | 0.07 | 0.54 | 0.99 | **0.77** | 0.75 | 0.30 | 1.00 |
|  | Water | 74.69 | 61.10 | 8 | 282 | 2.87 | 0.78 | 0.84 | 4.58 | 0.86 | 0.11 | 0.37 | 0.98 | 0.72 | 0.11 | 0.36 | 0.93 |
| *Bd-*positive | *A. obstetricans* | **26.08** | 13.60 | 11 | 51 | 2.06 | 0.72 | 0.37 | 3.94 | 0.71 | 0.16 | 0.13 | 0.96 | **0.69** | 0.29 | 0.1 | 1.00 |
|  | *B. spinosus* | **66.21** | 1.32 | 64 | 69 | 2.95 | 0.75 | 1.75 | 4.26 | 0.81 | 0.14 | 0.51 | 0.96 | **0.70** | 0.78 | 0.42 | 1.00 |
|  | *R. temporaria* | **110.68** | 40.39 | 73 | 210 | 1.88 | 0.85 | 0.89 | 4.22 | 0.67 | 0.16 | 0.37 | 0.97 | **0.41** | 0.20 | 0.18 | 0.98 |
|  | Biofilm | 470.00 | 92.33 | 45 | 470 | **4.95** | 0.71 | 1.35 | 4.95 | **0.92** | 0.09 | 0.53 | 0.98 | **0.73** | 0.14 | 0.27 | 0.98 |
|  | Water | 181.00 | 47.41 | 11 | 181 | 4.09 | 0.79 | 0.32 | 4.09 | 0.97 | 0.16 | 0.10 | 0.97 | 0.95 | 0.15 | 0.10 | 0.95 |

**Table S2.** Results of the ANOVA test computed between the diversity indexes of the three amphibian hosts and their environment. Benjamini-Hochberg correction was used to compute adjusted p-value. Ao = A. obstetricans, Bs = B. spinosus, Rt = R. temporaria, Bf = Biofilm and W = Water.

| **Tested index** | **Tested group** | **Diff** | **lwr** | **upr** | ***p.* adj.** | **signif.** |
| --- | --- | --- | --- | --- | --- | --- |
| ASV number | Bf-*Ao* | 220.64 | 206.01 | 235.27 | <0.01 | * |
|  | *Bs*-*Ao* | 37.88 | 4.62 | 71.15 | 0.02 | * |
|  | *Rt*-*Ao* | 103.15 | 86.99 | 119.32 | <0.01 | * |
|  | W-*Ao* | 44.86 | 29.35 | 60.38 | <0.01 | * |
|  | *Bs*-Bf | -182.76 | -216.73 | -148.78 | <0.01 | * |
|  | *Rt*-Bf | -117.49 | -135.06 | -99.92 | <0.01 | * |
|  | W-Bf | -175.78 | -192.75 | -158.80 | <0.01 | * |
|  | *Rt*-*Bs* | 65.27 | 30.60 | 99.93 | <0.01 | * |
|  | W-*Bs* | 6.98 | -27.39 | 41.34 | 0.98 | ns |
|  | W-*Rt* | -58.29 | -76.61 | -39.97 | <0.01 | * |
| Shannon | Bf-*Ao* | 2.07 | 1.86 | 2.27 | <0.01 | * |
|  | *Bs*-*Ao* | 0.74 | 0.28 | 1.20 | <0.01 | * |
|  | *Rt*-*Ao* | -0.15 | -0.37 | 0.08 | 0.37 | ns |
|  | W-*Ao* | 0.80 | 0.59 | 1.02 | <0.01 | * |
|  | *Bs*-Bf | -1.33 | -1.80 | -0.86 | <0.01 | * |
|  | *Rt*-Bf | -2.21 | -2.46 | -1.97 | <0.01 | * |
|  | W-Bf | -1.26 | -1.50 | -1.03 | <0.01 | * |
|  | *Rt*-*Bs* | -0.88 | -1.36 | -0.41 | <0.01 | * |
|  | W-*Bs* | 0.07 | -0.41 | 0.54 | 1.00 | ns |
|  | W-*Rt* | 0.95 | 0.70 | 1.20 | <0.01 | * |
| Simpson | Bf-*Ao* | 0.23 | 0.19 | 0.27 | <0.01 | * |
|  | *Bs*-*Ao* | 0.10 | 0.01 | 0.19 | 0.02 | * |
|  | *Rt*-*Ao* | -0.05 | -0.09 | -0.01 | 0.01 | * |
|  | W-*Ao* | 0.15 | 0.11 | 0.19 | <0.01 | * |
|  | *Bs*-Bf | -0.13 | -0.22 | -0.04 | <0.01 | * |
|  | *Rt*-Bf | -0.28 | -0.33 | -0.24 | <0.01 | * |
|  | W-Bf | -0.08 | -0.12 | -0.03 | <0.01 | * |
|  | *Rt*-*Bs* | -0.15 | -0.24 | -0.06 | <0.01 | * |
|  | W-*Bs* | 0.05 | -0.04 | 0.14 | 0.48 | ns |
|  | W-*Rt* | 0.20 | 0.15 | 0.25 | <0.01 | * |
| Evenness | Bf-*Ao* | 0.11 | 0.06 | 0.17 | <0.01 | * |
|  | *Bs*-*Ao* | 0.01 | -0.11 | 0.14 | 1.00 | ns |
|  | *Rt*-*Ao* | -0.25 | -0.31 | -0.19 | <0.01 | * |
|  | W-*Ao* | 0.07 | 0.01 | 0.13 | 0.01 | * |
|  | *Bs*-Bf | -0.10 | -0.22 | 0.03 | 0.19 | ns |
|  | *Rt*-Bf | -0.36 | -0.43 | -0.30 | <0.01 | * |
|  | W-Bf | -0.04 | -0.11 | 0.02 | 0.30 | ns |
|  | *Rt*-*Bs* | -0.26 | -0.39 | -0.14 | <0.01 | * |
|  | W-*Bs* | 0.06 | -0.07 | 0.18 | 0.75 | ns |
|  | W-*Rt* | 0.32 | 0.25 | 0.39 | <0.01 | * |

**Table S3.** Average relative abundance and percentage of positive samples for the 30 micro-eukaryotic genera dominating the amphibian tadpole skin samples. When unclassified, the first taxonomic level that could be classified is shown. Red shows highest relative abundance, green show lowest proportions. Ao = A. obstetricans, Bs = B. spinosus and Rt = R. temporaria

| **Genera** | **Average relative abundance** | | | **%age of positive samples** | | |
| --- | --- | --- | --- | --- | --- | --- |
|  | ***Ao*** | ***Bs*** | ***Rt*** | ***Ao*** | ***Bs*** | ***Rt*** |
| *Vorticella* | 54.11 | 42.33 | 64.09 | 98.31 | 95.83 | 97.83 |
| *Desmodesmus* | 2.06 | 2.08 | 2.59 | 77.29 | 70.83 | 69.57 |
| *Dinophyceae_unclassified_unclassified_unclassified* | 2.13 | 7.55 | 2.37 | 61.36 | 75 | 53.62 |
| *Peridiniales_unclassified_unclassified* | 2.95 | 0.48 | 2.35 | 20.34 | 20.83 | 22.46 |
| *Embryophyceae_unclassified_unclassified* | 1.52 | 2.06 | 2.19 | 69.83 | 100 | 65.94 |
| *Eukaryota_unclassified* | 0.64 | 0.57 | 1.79 | 67.8 | 91.67 | 54.35 |
| *Saprolegniales_unclassified* | 0.03 | 0.03 | 1.2 | 9.15 | 25 | 15.22 |
| *Sphaeropleales_unclassified_unclassified* | 1.16 | 0.42 | 1.02 | 50.51 | 79.17 | 57.25 |
| *Dothideomycetes_unclassified* | 0.83 | 1.89 | 0.94 | 57.63 | 100 | 54.35 |
| *Actinocephalidae_unclassified* | 0.07 | 0.09 | 0.79 | 11.53 | 8.33 | 7.25 |
| *Candida* | 0.02 | 0.31 | 0.74 | 10.85 | 45.83 | 8.7 |
| *Trichodinidae_unclassified* | 2.27 | 0.37 | 0.67 | 14.92 | 20.83 | 3.62 |
| *Fragilariales_unclassified_unclassified* | 0.88 | 1.44 | 0.62 | 54.58 | 75 | 58.7 |
| *Chrysosaccales_unclassified_unclassified* | 1.29 | 0.24 | 0.56 | 40.68 | 8.33 | 14.49 |
| *Chloromonas* | 0.09 | 0.31 | 0.46 | 12.54 | 29.17 | 18.12 |
| *Tremellomycetes_unclassified* | 0.33 | 0.9 | 0.45 | 39.32 | 79.17 | 37.68 |
| *Peritrichia_2_unclassified_unclassified* | 2.11 | 1.17 | 0.45 | 45.08 | 41.67 | 22.46 |
| *Davidiella* | 0.39 | 1.9 | 0.44 | 50.51 | 91.67 | 50 |
| *Euteratocephalus* | 0.01 | 0.04 | 0.43 | 5.08 | 8.33 | 5.07 |
| *Anurofeca* | 0.2 | 0.24 | 0.43 | 40 | 50 | 43.48 |
| *Chlorophyceae_unclassified_unclassified_unclassified* | 0.19 | 0.39 | 0.4 | 47.46 | 62.5 | 34.06 |
| *Rhodotorula* | 0.32 | 0.72 | 0.38 | 34.92 | 70.83 | 42.75 |
| *Sessilida_unclassified* | 0.32 | 2.41 | 0.38 | 33.9 | 37.5 | 21.74 |
| *Peridinium* | 0.4 | 0 | 0.34 | 9.15 | 0 | 7.97 |
| *Cryptococcus* | 0.29 | 0.65 | 0.33 | 37.63 | 83.33 | 34.78 |
| *Baldinia* | 0.08 | 1.48 | 0.33 | 4.75 | 16.67 | 9.42 |
| *Fungi_unclassified* | 0.24 | 0.39 | 0.31 | 51.19 | 75 | 54.35 |
| *Penicillium* | 0.41 | 2.33 | 0.29 | 20.68 | 45.83 | 18.12 |

**Table S4.** Average relative abundance and percentage of positive samples for the 30 micro-eukaryotic genera dominating the biofilm samples. When unclassified, the first taxonomic level that could be classified is shown. Red shows highest relative abundance, green show lowest proportions. Abundance of each genera in water samples is also showed. Bf = Biofilm and W = Water.

| **Genus** | **Average relative abundance** | | **%age of positive samples** | |
| --- | --- | --- | --- | --- |
|  | **Biofilm** | **Water** | **Biofilm** | **Water** |
| *Desmodesmus* | 18.12 | 2.80 | 96.81 | 41.03 |
| *Rhabdolaimus* | 7.58 | 0.15 | 79.37 | 12.18 |
| *Chaetonotus* | 5.72 | 0.53 | 87.83 | 30.13 |
| *Lepidodermella* | 4.62 | 0.29 | 60.32 | 14.10 |
| *Adineta* | 3.74 | 0.11 | 75.13 | 11.54 |
| *Cymbella* | 2.83 | 0.65 | 40.74 | 26.28 |
| *Eumonhystera* | 2.71 | 0.11 | 86.77 | 10.90 |
| *Navicula* | 2.20 | 0.81 | 64.02 | 26.92 |
| *Asulcocephalium* | 1.89 | 5.28 | 50.79 | 46.15 |
| *Borghiella* | 1.64 | 0.19 | 68.25 | 9.62 |
| *Anurofeca* | 1.50 | 0.47 | 60.32 | 22.44 |
| *Baldinia* | 1.32 | 3.27 | 20.63 | 21.79 |
| *Leptophrys* | 1.23 | 0.10 | 49.74 | 8.33 |
| *Stentor* | 1.21 | 0.46 | 40.74 | 17.95 |
| *Syncystis* | 1.00 | 0.08 | 37.04 | 10.90 |
| *Nannochloropsis* | 0.94 | 0.46 | 60.32 | 19.87 |
| *Rhizophydiales_unclassified_unclassified* | 0.90 | 0.64 | 73.54 | 36.54 |
| *Chloromonas* | 0.87 | 0.16 | 47.09 | 5.77 |
| *Spirogyra* | 0.81 | 0.06 | 19.05 | 6.41 |
| *Lagenidium* | 0.81 | 1.21 | 52.91 | 20.51 |
| *Mychonastes* | 0.68 | 0.79 | 66.14 | 17.95 |
| *Peridinium* | 0.67 | 8.00 | 24.87 | 37.18 |
| *Vorticella* | 0.66 | 1.90 | 53.97 | 50.00 |
| *Leidyana2* | 0.58 | 0.07 | 28.04 | 8.97 |
| *Vernalophrys* | 0.57 | 0.01 | 58.73 | 3.85 |
| *Petalomonas* | 0.56 | 0.00 | 43.92 | 0.64 |
| *Achnanthidium* | 0.55 | 0.38 | 28.57 | 17.31 |
| *Rhogostoma-lineage_unclassified* | 0.53 | 0.01 | 41.27 | 1.28 |
| *Ulothrix* | 0.48 | 0.01 | 4.76 | 0.64 |
| *Nassulida_unclassified* | 0.47 | 0.00 | 5.82 | 0.64 |

| **Genus** | **Average relative abundance** | |  | **%age of positive samples** | |
| --- | --- | --- | --- | --- | --- |
|  | **Biofilm** | **Water** |  | **Biofilm** | **Water** |
| Strobilidiidae_unclassified | 0.09 | 8.96 |  | 19.05 | 51.28 |
| Cryptomonas | 0.43 | 8.36 |  | 47.09 | 71.15 |
| Peridinium | 0.67 | 8.00 |  | 24.87 | 37.18 |
| Stephanodiscus | 0.38 | 5.30 |  | 17.99 | 27.56 |
| Asulcocephalium | 1.89 | 5.28 |  | 50.79 | 46.15 |
| Baldinia | 1.32 | 3.27 |  | 20.63 | 21.79 |
| Hydrurus | 0.17 | 3.24 |  | 16.40 | 13.46 |
| Mallomonas | 0.16 | 3.21 |  | 14.29 | 35.26 |
| Desmodesmus | 18.12 | 2.80 |  | 96.81 | 41.03 |
| Hexasterias | 0.01 | 2.58 |  | 5.82 | 39.74 |
| Novel-clade-10_ unclassified_ unclassified | 0.00 | 2.05 |  | 2.12 | 32.69 |
| Vorticella | 0.66 | 1.90 |  | 53.97 | 50.00 |
| Kathablepharidida_ unclassified_ unclassified | 0.01 | 1.54 |  | 1.06 | 31.41 |
| Lagenidium | 0.81 | 1.21 |  | 52.91 | 20.51 |
| Ichthyophthirius | 0.21 | 1.15 |  | 17.46 | 24.36 |
| Dinobryon | 0.02 | 1.14 |  | 4.76 | 17.95 |
| Chrysophyceae_Cluster-I_ unclassified_ unclassified | 0.00 | 1.12 |  | 2.12 | 22.44 |
| Plagioselmis | 0.00 | 1.02 |  | 1.06 | 15.38 |
| Halteria | 0.26 | 0.91 |  | 11.64 | 8.97 |
| Pseudopedinella | 0.00 | 0.85 |  | 0.53 | 27.56 |
| Urotricha | 0.02 | 0.83 |  | 3.70 | 23.72 |
| Navicula | 2.20 | 0.81 |  | 64.02 | 26.92 |
| Mychonastes | 0.68 | 0.79 |  | 66.14 | 17.95 |
| Chrysosaccus | 0.02 | 0.78 |  | 6.35 | 16.03 |
| Pelagostrombidium | 0.00 | 0.77 |  | 1.06 | 14.10 |
| Oligotrichida_ unclassified_ unclassified | 0.02 | 0.76 |  | 6.35 | 7.69 |
| MAST-12C | 0.14 | 0.76 |  | 44.44 | 42.95 |
| Pedinellales_ unclassified | 0.00 | 0.76 |  | 0.00 | 25.64 |
| Chrysochromulina | 0.00 | 0.75 |  | 0.53 | 16.67 |
| Choricystis | 0.02 | 0.71 |  | 6.35 | 3.85 |

**Table S5.** Average relative abundance and percentage of positive samples for the 30 micro-eukaryotic genera dominating the water samples. When unclassified, the first taxonomic level that could be classified is shown. Red shows highest relative abundance, green show lowest proportions. Abundance of each genera in biofilm samples is also showed. Bf = Biofilm and W = Water.

**Table S6.** Results of the Wilcoxon test performed between the abundance of putative anti-Bd genera in the amphibian skin, the biofilm and the water samples, according to the Bd infectious status. Benjamini-Hochberg correction was performed to compute adjusted p-value. Ao = A. obstetricans, Bs = B. spinosus, Rt = R. temporaria, Bf = Biofilm and W = Water.

| **Tested index** | **Tested group** | **Diff** | **lwr** | **upr** | ***p.* adj.** | **signif.** |
| --- | --- | --- | --- | --- | --- | --- |
| Putative anti-*Bd* fungi | Bf*-Ao* | -0.59 | -1.49 | 0.31 | 0.38 | ns |
|  | *Bs-Ao* | -0.54 | -2.44 | 1.37 | 0.94 | ns |
|  | *Rt-Ao* | -0.57 | -1.80 | 0.67 | 0.72 | ns |
|  | W*-Ao* | -0.60 | -1.54 | 0.35 | 0.42 | ns |
|  | *Bs-*Bf | 0.05 | -1.86 | 1.96 | 1.00 | ns |
|  | *Rt-*Bf | 0.03 | -1.21 | 1.27 | 1.00 | ns |
|  | W*-*Bf | 0.00 | -0.96 | 0.95 | 1.00 | ns |
|  | *Rt-Bs* | -0.03 | -2.12 | 2.06 | 1.00 | ns |
|  | W*-Bs* | -0.06 | -1.99 | 1.87 | 1.00 | ns |
|  | W*-Rt* | -0.03 | -1.31 | 1.24 | 1.00 | ns |
| *Ciliata* | Bf*-Ao* | -46.09 | -53.16 | -39.01 | <0.01 | * |
|  | *Bs-Ao* | -16.23 | -31.18 | -1.27 | 0.03 | * |
|  | *Rt-Ao* | -0.67 | -10.35 | 9.01 | 1.00 | ns |
|  | W*-Ao* | -34.54 | -41.98 | -27.11 | <0.01 | * |
|  | *Bs-*Bf | 29.86 | 14.87 | 44.85 | <0.01 | * |
|  | *Rt-*Bf | 45.42 | 35.68 | 55.15 | <0.01 | * |
|  | W*-*Bf | 11.54 | 4.04 | 19.05 | <0.01 | * |
|  | *Rt-Bs* | 15.56 | -0.82 | 31.94 | 0.07 | ns |
|  | W*-Bs* | -18.32 | -33.48 | -3.16 | 0.01 | * |
| *Rotifera* | Bf*-Ao* | -33.87 | -43.88 | -23.87 | <0.01 | * |
|  | *Bs-Ao* | 4.35 | 2.96 | 5.75 | <0.01 | * |
|  | *Rt-Ao* | 0.64 | -2.31 | 3.59 | 0.98 | ns |
|  | W*-Ao* | -0.12 | -2.03 | 1.79 | 1.00 | ns |
|  | *Bs-*Bf | 2.93 | 1.47 | 4.40 | <0.01 | * |
|  | *Rt-*Bf | -3.71 | -6.67 | -0.75 | 0.01 | * |
|  | W*-*Bf | -4.47 | -6.39 | -2.55 | <0.01 | * |
|  | *Rt-*Bf | -1.42 | -2.90 | 0.06 | 0.07 | ns |
|  | W*-Bs* | -0.77 | -4.00 | 2.47 | 0.97 | ns |
|  | W*-Rt* | 2.29 | -0.70 | 5.28 | 0.22 | ns |
| Putative anti-*Bd Rotifera* and *Ciliata* | Bf*-Ao* | 0.13 | 0.05 | 0.20 | <0.01 | * |
|  | *Bs-Ao* | -0.03 | -0.19 | 0.13 | 0.99 | ns |
|  | *Rt-Ao* | 0.03 | -0.08 | 0.13 | 0.96 | ns |
|  | W*-Ao* | 0.08 | -0.01 | 0.16 | 0.08 | ns |
|  | *Bs-*Bf | -0.16 | -0.32 | 0.01 | 0.07 | ns |
|  | *Rt-*Bf | -0.10 | -0.21 | 0.01 | 0.08 | ns |
|  | W*-*Bf | -0.05 | -0.13 | 0.03 | 0.47 | ns |
|  | *Rt-*Bf | 0.06 | -0.12 | 0.24 | 0.91 | ns |
|  | W*-Bs* | 0.11 | -0.06 | 0.27 | 0.41 | ns |
|  | W*-Rt* | 0.05 | -0.06 | 0.16 | 0.72 | ns |

**Table S7.** Results of the Wilcoxon test performed between the diversity indexes of the amphibian skin, the biofilm and the water samples, according to the Bd infectious status. Benjamini-Hochberg correction was performed to compute adjusted p-value. Ao = A. obstetricans, Bs = B. spinosus, Rt = R. temporaria, Bf = Biofilm and W = Water.

|  | **Diversity indexes tested** | **Compartment tested** | **Degree of Freedom** | **W value** | ***p* adj.** | **Significance** |
| --- | --- | --- | --- | --- | --- | --- |
| **Hosts** | ASV number | *Ao Bd - ~ Bd+* | 83 | 9,180.00 | <0.01 | * |
|  |  | *Bs Bd - ~ Bd+* | 9 | 105.00 | <0.01 | * |
|  |  | *Rt Bd - ~ Bd+* | 22 | 300.00 | <0.01 | * |
|  | Shannon | *Ao Bd - ~ Bd+* | 83 | 6,472.00 | 0.88 | ns |
|  |  | *Bs Bd - ~ Bd+* | 9 | 91.00 | 0.24 | ns |
|  |  | *Rt Bd - ~ Bd+* | 22 | 857.50 | 0.96 | ns |
|  | Simpson | *Ao Bd - ~ Bd+* | 83 | 6,795.00 | 0.62 | ns |
|  |  | *Bs Bd - ~ Bd+* | 9 | 82.00 | 0.50 | ns |
|  |  | *Rt Bd - ~ Bd+* | 22 | 937.00 | 0.54 | ns |
|  | Evenness | *Ao Bd - ~ Bd+* | 83 | 9,180.00 | <0.01 | * |
|  |  | *Bs Bd - ~ Bd+* | 9 | 105.00 | <0.01 | * |
|  |  | *Rt Bd - ~ Bd+* | 22 | 300.00 | <0.01 | * |
| **Environment** | ASV number | Bf *Bd - ~ Bd+* | 50 | 3,635.50 | 0.09 | ns |
|  |  | W *Bd - ~ Bd+* | 40 | 2,260.50 | 0.37 | ns |
|  | Shannon | Bf *Bd - ~ Bd+* | 46 | 4,116.00 | <0.01 | * |
|  |  | W *Bd - ~ Bd+* | 41 | 2,319.00 | 0.28 | ns |
|  | Simpson | Bf *Bd - ~ Bd+* | 15 | 3,969.50 | 0.01 | * |
|  |  | W *Bd - ~ Bd+* | 23 | 2,277.00 | 0.34 | ns |
|  | Evenness | Bf *Bd - ~ Bd+* | 34 | 3,727.50 | 0.05 | * |
|  |  | W *Bd - ~ Bd+* | 29 | 2,261.50 | 0.37 | ns |

**Table S8.** Results of the Wilcoxon test computed between the relative abundance of putative anti-Bd fungi, Ciliata and Rotifera. Benjamini-Hochberg correction was performed to compute adjusted p-value. Ao = A. obstetricans, Bs = B. spinosus, Rt = R. temporaria, Bf = Biofilm and W = Water.

| **Tested index** | **Tested group** | **Degree of Freedom** | **W value** | **Adjusted p-value** | **Significance** |
| --- | --- | --- | --- | --- | --- |
| Putative anti-*Bd* fungi | *Ao Bd - ~ Bd+* | 113.00 | 0.45 | 0.32 | ns |
|  | *Bs Bd - ~ Bd+* | 9.00 | 45.00 | 0.04 | * |
|  | *Rt Bd - ~ Bd+* | 113.00 | 2.00 | 0.03 | * |
|  | Bf *Bd - ~ Bd+* | 164.00 | 3,456.00 | <0.01 | * |
|  | W *Bd - ~ Bd+* | 90.00 | 2,304.00 | <0.01 | * |
| *Ciliata* | *Ao Bd - ~ Bd+* | 54.00 | 5,046.00 | <0.01 | * |
|  | *Bs Bd - ~ Bd+* | 9.00 | 34.00 | 0.03 | * |
|  | *Rt Bd - ~ Bd+* | 20.00 | 142.50 | <0.01 | * |
|  | Bf *Bd - ~ Bd+* | 56.00 | 3,597.00 | 0.18 | ns |
|  | W *Bd - ~ Bd+* | 47.00 | 2,271.00 | 0.62 | ns |
| *Rotifera* | *Ao Bd - ~ Bd+* | 54.00 | 4,130.50 | 0.57 | ns |
|  | *Bs Bd - ~ Bd+* | 9.00 | 68.00 | 0.18 | ns |
|  | *Rt Bd - ~ Bd+* | 20.00 | 395.00 | 0.68 | ns |
|  | Bf *Bd - ~ Bd+* | 56.00 | 2,922.00 | 0.16 | ns |
|  | W *Bd - ~ Bd+* | 47.00 | 2,640.00 | 0.14 | ns |
| Putative anti-*Bd Rotifera* and *Ciliata* | *Ao Bd - ~ Bd+* | 7.00 | 3,570.00 | 0.03 | * |
|  | *Bs Bd - ~ Bd+* | 4.00 | 70.00 | 1.00 | ns |
|  | *Rt Bd - ~ Bd+* | 1.00 | 360.00 | 0.91 | ns |
|  | Bf *Bd - ~ Bd+* | 31.00 | 3,088.50 | 0.71 | ns |
|  | W *Bd - ~ Bd+* | 10.00 | 2,065.00 | 0.52 | ns |

**Table S9.** Percentage of contribution of biofilm and water samples in the building of micro-eukaryotic communities of amphibian skin inferred by the Bayesian SourceTracker analysis. Bold font shows significant differences for the same amphibian tadpole species between Bd-negative and Bd-positive lakes (Wilcoxon test with Benjamini-Hochberg correction). Contrib. = contribution, SE = Standar Error.

| **Lake *Bd* status** | **Host species** | **Biofilm contrib. ± SE** | **Water contrib. ± SE** | **Unknown**  **contrib. ± SE** |
| --- | --- | --- | --- | --- |
| All | *A. obstetricans* | 14.30 ± 0.14 | 14.55 ± 1.58 | 71.15 ± 2.02 |
|  | *B. spinosus* | 20.60 ± 3.35 | 16.17 ± 4.27 | 63.23 ± 4.81 |
|  | *R. temporaria* | 19.09 ± 2.94 | 19.52 ± 4.16 | 61.39 ± 4.57 |
| Positive | *A. obstetricans* | 12.25 ± 1.52 | 14.55 ± 1.85 | 73.20 ± 2.24 |
|  | *B. spinosus* | **27.72 ± 4.27** | 10.52 ± 4.91 | 61.76 ± 5.88 |
|  | *R. temporaria* | **37.06 ± 5.63** | 10.84 ± 5.65 | 53.48 ± 5.34 |
| Negative | *A. obstetricans* | 17.52 ± 2.79 | 15.67 ± 3.18 | 66.81 ± 4.03 |
|  | *B. spinosus* | **10.62 ± 3.45** | 24.08 ± 8.89 | 65.30 ± 8.04 |
|  | *R. temporaria* | **9.99 ± 1.89** | 9.46 ± 3.06 | 79.17 ± 4.55 |

**Table S10.** Results of the ANOVA test performed between the percentage of contribution from water and biofilm in the building of amphibian skin micro-eukaryotic communities. Benjamini-Hochberg correction was performed to compute adjusted p-value. Ao = A. obstetricans, Bs = B. spinosus, Rt = R. temporaria, Bf = Biofilm and W = Water.

| **Tested group** | **Diff** | **Lwr** | **upr** | ***p.* adj.** |
| --- | --- | --- | --- | --- |
| *Ao*_W *~ Ao*_Bf | 0.00 | -0.06 | 0.07 | 1.00 |
| *Bs*_Bf *~ Ao*_Bf | 0.06 | -0.07 | 0.19 | 0.79 |
| *Bs*_W *~ Ao*_Bf | 0.02 | -0.12 | 0.15 | 1.00 |
| *Rt*_Bf *~ Ao*_Bf | 0.06 | -0.04 | 0.15 | 0.49 |
| *Rt*_W *~ Ao*_Bf | -0.04 | -0.14 | 0.05 | 0.78 |
| *Bs*_Bf *~ Ao*_W | 0.06 | -0.08 | 0.19 | 0.83 |
| *Bs*_W *~ Ao*_W | 0.01 | -0.12 | 0.14 | 1.00 |
| *Rt*_Bf *~ Ao*_W | 0.05 | -0.04 | 0.15 | 0.56 |
| *Rt*_W *~ Ao*_W | -0.05 | -0.14 | 0.05 | 0.72 |
| *Bs*_W *~ Bs*_Bf | -0.04 | -0.22 | 0.13 | 0.98 |
| *Rt*_Bf *~ Bs*_Bf | 0.00 | -0.15 | 0.15 | 1.00 |
| *Rt*_W *~ Bs*_Bf | -0.10 | -0.25 | 0.05 | 0.37 |
| *Rt*_Bf *~ Bs*_W | 0.04 | -0.11 | 0.19 | 0.96 |
| *Rt*_W *~ Bs*_W | -0.06 | -0.21 | 0.09 | 0.87 |

**Table S11.** Results of the Wilcoxon test performed between the percentage of contribution from water and biofilm in the building of amphibian skin micro-eukaryotic communities according to the Bd infectious status of lake. Benjamini-Hochberg correction was performed to compute adjusted p-value. Ao = A. obstetricans, Bs = B. spinosus, Rt = R. temporaria, Bf = Biofilm and W = Water.

| **Amphibian host tested** | **Compartment** | **Degree of Freedom** | **W value** | **Adjusted p-value** | **Significance** |
| --- | --- | --- | --- | --- | --- |
| *A. obstetricans* | *Bf Bd +~ Bf Bd -* | 67 | 4,112.00 | 0.42 | ns |
|  | *W Bd +~ W Bd -* | 67 | 3,471.00 | 0.31 | ns |
| *B. spinosus* | *Bf Bd +~ Bf Bd -* | 9 | 26.00 | <0.01 | * |
|  | *W Bd +~ W Bd -* | 9 | 74.00 | 0.60 | ns |
| *R. temporaria* | *Bf Bd +~ Bf Bd -* | 20 | 130.50 | <0.01 | * |
|  | *W Bd +~ W Bd -* | 20 | 336.50 | 0.43 | ns |

**Table S12.** Features of each lake in which skin micro-eukaryotic microbiota were sampled from amphibian tadpoles. Number of amphibian samples (Nsamples) considered for the metabarcoding analyze is indicated by compartment and species. Ao = A. obstetricans, Bs = B. spinosus, Rt = R. temporaria, Bf = Biofilm and W = Water.

| **Lakes** | ***Bd* infectious status** | **Longitude** | **Latitude** | **Lake area (hectares)** | **Altitude (meters)** | ***Ao*** | ***Bs*** | ***Rt*** | **Bf** | **W** |
| --- | --- | --- | --- | --- | --- | --- | --- | --- | --- | --- |
| Acherito | positive | -0.71 | 42.88 | 7.46 | 1,880 | 32 | 4 |  | 9 | 8 |
| Alate | negative | 1.41 | 42.78 | 2.13 | 1,865 |  |  | 2 | 13 | 8 |
| Ansabere | positive | -0.71 | 42.89 | 0.21 | 1,850 | 21 | 10 | 10 | 9 | 8 |
| Arbu | negative | 1.44 | 42.82 | 5.01 | 1,737 |  |  | 10 | 11 | 7 |
| Arlet | positive | -0.61 | 42.84 | 3.46 | 1,974 | 15 |  |  | 11 | 8 |
| Ayes | positive | 1.06 | 42.84 | 1.87 | 1,714 | 40 |  | 12 | 10 | 9 |
| Bellonguere | negative | 1.06 | 42.84 | 0.17 | 1,907 | 13 |  | 20 | 12 | 9 |
| Bethmale | negative | 1.08 | 42.86 | 2.91 | 1,063 |  | 5 | 5 | 6 | 10 |
| Embarrat | negative | -0.19 | 42.84 | 0.01 | 2,180 | 22 |  | 9 | 7 | 5 |
| Fache-1 | negative | -0.21 | 42.80 | 0.01 | 2,522 | 12 |  | 3 | 5 | 6 |
| Gourg-de-Rabas | negative | 0.15 | 42.85 | 1.33 | 2,422 | 6 |  | 2 | 10 | 8 |
| Fache-2 | negative | -0.22 | 42.81 | 0.87 | 2,422 | 16 |  |  | 10 | 8 |
| Labant | negative | 1.39 | 42.78 | 0.46 | 1,600 |  |  | 7 | 8 | 8 |
| Lhurs | positive | -0.7 | 42.92 | 3.59 | 1,697 | 26 |  |  | 9 | 7 |
| Lhurs (pond) | positive | -0.63 | 42.86 | 0.001 | 1,880 |  |  | 9 |  |  |
| Madamete-Haut | changing | 0.14 | 42.86 | 0.31 | 2,374 |  |  | 17 | 9 | 8 |
| Mort | negative | 1.42 | 42.76 | 0.86 | 1,651 |  | 5 |  | 12 | 6 |
| Paradis | changing | -0.16 | 42.85 | 0.42 | 1,609 | 23 |  | 8 | 9 | 8 |
| Pecheur | negative | 0.15 | 42.87 | 0.59 | 2,310 |  |  | 5 | 9 | 8 |
| Puits d'Arrious | positive | -0.63 | 42.86 | 0.26 | 1,880 | 41 |  |  | 9 | 8 |
| Puits d’Arrious (pond) | positive | -0.63 | 42.86 | 0.01 | 1,880 |  |  | 5 |  |  |
| Vallon | negative | -0.19 | 42.84 | 0.05 | 2,215 | 27 |  | 15 | 9 | 6 |
